# Holistic analysis of urban water systems in the Greater Cincinnati region: (2) resource use profiles by emergy accounting approach

**DOI:** 10.1016/j.wroa.2018.100012

**Published:** 2018-11-22

**Authors:** Sam Arden, Xin (Cissy) Ma, Mark Brown

**Affiliations:** a UF Center for Environmental Policy, 102 Phelps Laboratory, University of Florida, P.O. Box 116530, Gainesville, FL, 32611-6350, USA; b US EPA ORD, National Risk Management Research Laboratory, 26 West Martin Luther King Drive, Cincinnati, OH, 45268, USA

**Keywords:** Emergy, Integrated urban water management, Sustainability, System analysis, Resource efficiency

## Abstract

With increasing populations, mounting environmental pressures and aging infrastructure, urban water and wastewater utilities have to make investment decisions limited by both economic and environmental constraints. The challenges facing urban water systems can no longer be sustainably solved by traditional siloed water management approaches. A central premise of contemporary urban water management paradigms is that in order for urban water systems to be more sustainable and economical, an improvement in resource use efficiency at system level must be achieved. This study provides a quantification of the total resource use of a typical urban water system exemplified in Greater Cincinnati region from raw water extraction for drinking water to wastewater treatment and discharge, providing a better understanding of resource expenditure distributions within the system and a necessary benchmark to which future system improvements can be compared. The emergy methodology was used so that the total environmental work required to produce disparate system inputs could be expressed using a common unit. The results were compared to the concurring life cycle assessment (LCA) and life cycle costing (LCC) results of the same system. Emergy results highlight drinking water treatment and drinking water distribution as two resource-intensive stages, with energy for pumping and chemicals for conditioning representing the greatest inputs to the former and energy for pumping and metals for piping representing the greatest inputs to the latter. For wastewater collection and treatment stages, aeration and sludge handling were identified as the highest emergy unit processes, mostly due to energy use. Comparison with LCA results substantiate the environmental concerns associated with energy use in the drinking water treatment and distribution stages but indicate that environmental burdens associated with infrastructure are more dependent upon upstream resource use rather than downstream environmental impact. Results from emergy, LCA and LCC point towards aeration and sludge handling as two unit processes on the wastewater side that are particularly costly and environmentally impactful. Results in total are used to suggest alternative strategies that can alleviate identified environmental burdens and economic costs.

## Highlights

• Centralized public water supply is the most resource intensive urban water service.
• Distribution is particularly resource intensive owing to energy and infrastructure.
• Most resource intense wastewater unit processes are aeration and sludge handling.
• Emergy and LCA together can better quantify full environmental burdens.

## Graphical abstract

**Figure:**
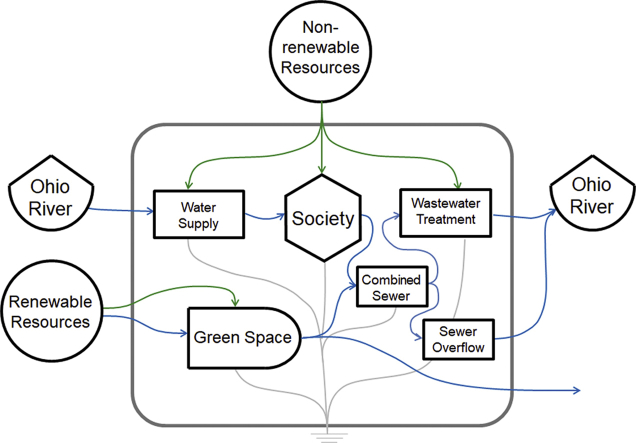

## 1 Introduction

Urban water challenges in industrialized nations are no longer solely comprised of the low-cost provision of supply, sanitation and drainage services. As populations increase, energy and water resources become more scarce and ecological impacts mount, urban water resource managers must also take into account factors such as total resource use and environmental impacts of investment decisions. To do so, managers must have a holistic understanding of current urban water systems and search for system-level, integrated solutions that maximize public utility operation (Alliance, 2017; Hering et al., 2013; Luthy, 2013). These themes are echoed in recently developed management approaches including Sustainable Urban Water Management (SUWM) (Larsen and Gujer, 1997; Marlow et al., 2013), Integrated Urban Water Management (IUWM) and Water Sensitive Urban Design (WSUD) (Wong, 2006). A central theme of each approach is that in order for urban water systems (UWS, here broadly referring to the infrastructure associated with the provision of supply, sanitation and drainage services in urban areas) to become more sustainable, an improvement in overall resource use efficiency is necessary (Marlow et al., 2013). Thus, a necessary first step is the quantification of the total resource use of the existing UWS.

Emergy analysis is a method used to quantify and compare different resource inputs using a common unit, providing a unique, broad and inclusive measure of total resource use of a system. In contrast to traditional economic accounting, which primarily accounts for the human labor required to make a product or service, emergy also accounts for the work done by nature to produce the natural capital (e.g. water, energy, minerals, etc.) upon which those products or services depend, thus providing a direct accounting of the full resource costs.

Previous studies have used emergy analysis to evaluate different components of the urban water system including different drinking water treatment plants (DWTP) (Arbault et al., 2013; Buenfil, 2001; Pulselli et al., 2011), conventional wastewater treatment plants (WWTP) (Geber and Bjorklund, 2001; Nelson, 1998; Siracusa and La Rosa, 2006; Vassallo et al., 2009) and alternatives to conventional WWTPs such as anaerobic digesters (Moss et al., 2014) and treatment wetlands (Arias and Brown, 2009; Geber and Bjorklund, 2001; Nelson, 1998; Siracusa and La Rosa, 2006). While quantifying the resource costs of water treatment using different approaches, which are shown to be site specific and highly dependent upon the locally-demanded level of service, these studies also provided significant advances to the emergy methodology. For example, Buenfil (2001) provided a comprehensive evaluation of water supply alternatives and used the emergy to money ratio to show that potable water is highly economically undervalued. Arbault et al. (2013), through the use of emergy-based indicators, showed that DWTPs are ‘rather blind to economic markets’ and exert a low pressure on local non-renewable resources at the expense of imported non-renewable resources. In studies comparing traditional WWTPs to alternative treatment approaches such as constructed wetlands, study objectives varied but were loosely based on the idea that in order to improve the sustainability of wastewater treatment, treatment systems should use more renewable resources and less total resources. To that end, Arias and Brown (2009) showed that when land area is available and wastewater flows aren't very large, constructed wetlands provide greater value than conventional WWTPs in terms of performance, cost and resource utilization. Siracusa and La Rosa (2006) showed that treating WWTP effluent with a constructed wetland and beneficially reusing the final effluent in dry, rural agricultural areas conferred a reduction in net resource use compared to full treatment in a WWTP and discharge to a river. Lastly, Geber and Bjorkland found that when holding level of service (in this case phosphorus removal) constant, the total resource inputs required for treatment using a tertiary WWTP, secondary WWTP + constructed wetland, and natural wetland only, were strikingly similar (Geber and Bjorklund, 2001).

Though useful, past studies have largely fallen short in considering an individual treatment system's interaction with the next larger system, i.e. the urban water systems. Although drinking water or wastewater treatment is a system itself, but only a subsystem when the entire urban water system is considered. For example, water reuse at the neighborhood scale may be more resource intensive than a centralized WWTP, however if it offsets potable demand and reduces the piping infrastructure requirements, there may be a net improvement to overall UWS efficiency, not to mention the greater resiliency conferred through a lessened dependence on raw water import. Unfortunately, examples of such holistic analyses remain rare due to the inherent variability in different systems as well as the lack of a suitable framework and common unit of measure to assess the complex interactions in a clear and concise way (Burn et al., 2012; Hester and Little, 2013). Based on those knowledge of subsystems the more comprehensive evaluations of the next larger system (urban water system) become more important if overall system efficiency and sustainability are the goals of urban water management. After all, the system is more than the sum of its part (Xue et al., 2015; Ma et al., 2015). Emergy provides the unique common measure equipped to explore the behavior of a system as a whole and the interactions between subcomponents can be observed and optimized and its sustainability can be assessed. Often without looking at the next larger system, it limits our understanding of the organization and relative (in)efficiencies of the current system. As the foundation of emergy theory and evaluation methods, *Maximum Empower Principle* states that all self-organizing systems tend to maximize their rates of emergy use or empower, and those system that maximize empower will prevail (Brown and Herendeen, 1996; Odum, 1996). In other words, prevailing systems tend to produce a maximum power output, and for this purpose operate at optimal efficiency rather than at maximum efficiency. Emergy method offers an alternative perspective to the historically narrow attempt to equate ‘sustainability’ with ‘use fewer resources’. In the context of societal sub-systems (i.e. the UWS), implications can be thought of in terms of nested feedbacks. Sub-systems that feed into or back upon the next larger system beget stronger, more competitive systems that are able to reinforce resource intake and direct net resource savings into the development of more organized, sustainable states. Using emergy analysis to evaluate the degree to which resources flow through or are fed back could be a powerful way of gauging the contribution of alternative UWS configurations to the competitiveness, and thus sustainability, of the larger societal system.

Lastly, while the complexity of the UWS warrants a systems approach, its multidimensional nature warrants the use of multiple metrics to avoid externalization of impacts (Mayer, 2008; Xue et al., 2015). Emergy and Life Cycle Assessment (LCA) are two integrated assessment metrics that have been used in parallel or in hybrid in many sustainability evaluations of regional or product systems. Emergy is a donor-perspective concept while LCA is a receiver/user-perspective one. Emergy captures the natural capital and ecosystem contribution to a system (regional or product-based). It focuses on total resource use. For example, for phosphorus and its derivative production, emergy includes how much work the nature has to invest to produce phosphorus ore that has market values in technosphere. In LCA, the system boundary of phosphorus product starts with the technosphere mining process, but does not include the embedded values in phosphorus rock. However, besides the technological inputs, LCA includes environmental emissions as part of life cycle inventory. The environmental impacts are the focus of LCA. A methodology that uses multiple metrics may compensate each other for weakness and provide better insights of the complexity of the system performance. (Ingwersen, 2011; Raugei et al., 2014; Ulgiati et al., 2006). Due to the complementary natures of the two tools, some researchers explore the hybrid approach such as Emergy Life Cycle Assessment by combining the features of emergy with LCA (Bakshi, 2000; Baral and Bakshi, 2010; Ulgiati et al., 2007; Pizzigallo et al., 2008; Rugani et al., 2011; Rugani and Benetto, 2012). Or the two tools are used as complementary metrics to capture multi-facets of an environmental system that are relevant to sustainability (Hopton et al., 2010; Ingwersen et al., 2014; Arbault et al., 2013).

The comparison of the two metrics may provide the insights to maximize system efficiency while minimize environmental impacts. This study provides the first emergy analysis of a complete UWS from source water abstraction to wastewater discharge, using real data from the greater Cincinnati area. It is a companion paper to Xue et al. (2018) which provides an LCA and Life Cycle Costing (LCC) of the same system. Results are first presented at the DWTP and WWTP scale, showing the total resource requirements of each unit process and then discussed in comparison with LCA and LCC findings. Then emergy flows are shown at the UWS scale, using a subwatershed located within the service areas of the treatment plants to explore the nesting relationship of the built environment within its supporting natural environment.

## 2 Methods

### 2.1 Cincinnati water treatment plants

The two treatment plants studied are the Greater Cincinnati Water Works (GCWW) Richard Miller Water Treatment Plant (DWTP) and the Metropolitan Sewer District of Greater Cincinnati (MSD) Mill Creek Wastewater Treatment Plant (WWTP), both located in Cincinnati, Ohio. For each plant, an LCA and operational cost assessment at the unit process level was performed following the International Organization for Standardizations (ISO) 140,140 series (USEPA and Development, 2014a, USEPA and Development, 2014b). This study utilized the Life Cycle Inventories (LCI) using operational data from 2011. The DWTP LCI included the unit processes in the source water acquisition, water treatment train, and distribution network to the consumer. The WWTP LCI evaluated the unit processes including sewer collection network, treatment train, effluent discharge, and sludge disposal. For infrastructure components, inputs were annualized over the assumed lifetime of the component (Table 1). Both LCIs include infrastructure and operational inputs. General plant parameters are given in Table 1.

**Table 1:** General parameters for Greater Cincinnati Water Works (GCWW) supply system and Municipal Sewer District of Greater Cincinnati (MSDGC) sanitation system.

Parameter	Unit	GCWW Supply	MSDGC Sanitation
Year of Inventory		2011	2011
Year Plant Built		1906	1959
Annual Volume Delivered/Discharged	MGD	89	114
Annual Volume Delivered/Discharged	m³	123,560,247	157,615,342
Distribution/Collection Network Piping	mile	3,135	1,697
Distribution/Collection Network Piping	km	5,045	2,731
Geographic Area Served	km^2^	–	344
Number of People Served	ppl.	830,000	518,000
Assumed Building, Tank and Pipe Lifetime	yr	100	100
Assumed Pump and Motor Lifetime	yr	25	25

The DWTP (Fig. S1) has a capacity of 240 million gallons per day (MGD) and supplies water for the greater Cincinnati region and part of Kentucky. On average in 2011 it processed 106 MGD of source water from the Ohio River and delivered 89 MGD to consumers, with the remaining 17 MGD attributed to losses in the distribution system. Once source water is pumped to the plant, suspended solids are removed through coagulation with aluminum sulfate and gravity settling. The resulting sludge is thickened and disposed back to Ohio river, while the supernatant proceeds through sand filtration to remove additional solids. Following filtration, organics and adsorbable micro-pollutants are removed using granular activated carbon (GAC), which has to be periodically regenerated on-site. Prior to distribution, the water is conditioned to adjust pH, disinfected, and fluorinated. Chlorine residuals are maintained in the distribution system (USEPA, 2014b).

The WWTP (Fig. S2) has a nominal capacity of 120 MGD and a maximum capacity of 360 MGD to accommodate high flows from the combined sewer during wet weather events. During high flow events, the flow can exceed the capacity of the WWTP and the excess combined sewage bypasses to nearby Mill Creek. During non-wet weather events, typical wastewater are from households, industry and stream baseflow. Lift station pumping is necessary along the collection system, however the majority of transport energy is gravity-based since the WWTP sits at the bottom of the sewershed. At the WWTP, the treatment train includes a screening step for large and settle-able debris, primary sedimentation for suspended solid removal, secondary treatment of dissolved organics using an aerobic activated sludge process, secondary clarifiers to settle flocs, and disinfection prior to discharge. Sludge from primary and secondary treatment steps is thickened, dewatered, incinerated and the ash is disposed in a landfill.

### 2.2 Lick Run UWS

In order to perform an emergy analysis of a complete UWS, a sub-watershed located within the service area boundaries of the assessed DWTP and WWTP was selected (DWTP and WWTP total service areas were not used directly as they are not identical, only overlapping). Lick Run is a 2,900 acre sub-watershed of the Lower Mill Creek watershed in Cincinnati, OH, which sits on the north bank of the Ohio River. It has become the focal point of a larger effort by MSD to reduce wet weather sewage discharges as it has the largest combined sewer overflow (CSO) in MSD's service area, representing a quarter of the total wastewater flow generated within Lick Run (MSDGC, 2009). A number of reports have been written documenting existing conditions and proposed solutions (USEPA, 2011), from which basin characteristics and hydrologic flows (basin area, % imperviousness, % vegetated, annual precipitation, annual evapotranspiration) were derived (see Table S9 for calculations and sources). Since Lick Run is a sub-watershed, resource flows associated with the DWTP and WWTP were down-scaled according to the population of Lick Run (13,750) relative to the service population of both treatment plants. Treated drinking water allocation to indoor potable, indoor nonpotable and outdoor use according to Mayer et al. (1999).

### 2.3 Emergy analysis

Emergy is defined as the available energy of one form that is used up in transformations directly and indirectly to make a product or service (Odum, 1996). Grounded in thermodynamics and general system theory, it accounts for quality differences between forms of resources and energy using a single, common unit of measure (solar emjoules, sej). The general application of the method for inputs to a process or system is demonstrated in Equation (1): for each input flow of material, energy or labor (*x*_*i*_), a specific quality factor Unit Emergy Value (*UEV*_*i*_) is applied, resulting in an emergy value for each pathway. UEVs are expressed in units of sej (solar emjoules) per mass, volume, energy or dollars (depending on the particular flow, *x*_*i*_).

(Eq. 1) $$Emergy=\;\overset{i=n}{\underset{i=1}{\sum }}UEV_{i}\text{*}x_{i}$$

Application of Equation (1) to each individual input allows for the quantification of total emergy input to a process or system (e.g. a drinking water treatment plant). Conversely, if the objective is to obtain a quality factor, or UEV, for the output of a process or system (e.g. the treated water from the drinking water treatment plant), a rearranged version of Equation (1) would be used where individual emergy inputs are summed then divided by the output quantity *x*.

For this study, both approaches were utilized. For inputs to the evaluated components of the study system, including treatment plants and pipe networks, UEVs were obtained from the literature. For emergy flows along pathways in the subsequent Lick Run analysis, the inversion of Equation (1) was used to calculate, for example, the UEV of treated drinking water provided to a household. All UEVs used, calculated and cited hereafter are referenced to the 1.20 E25 sej/yr global emergy baseline (Brown et al., 2016). UEV library and emergy calculation tables are provided in the supplemental information.

## 3 Results

Table 2 shows the results of the emergy analysis for the Cincinnati DWTP and WWTP. It requires 1.8E+12 sej of resource inputs to provide 1 m^3^ of potable water to a Cincinnati consumer, nearly twice as much as the 9.1E+11 sej required to collect and treat 1 m^3^ of combined wastewater. For drinking water treatment (including infrastructure, no distribution, no source water), 8.8E+11 sej/m^3^ is within the range of comparable results for drinking water treatment from the literature of 4.0E+11 to 11E+11 sej/m^3^ (Arbault et al., 2013; Buenfil, 2001; Pulselli et al., 2011). For wastewater treatment without collection, 7.3E+11 sej/m^3^ is required by the MSDGC system. This is also comparable, though slightly less than past studies, which showed a range of 6.9E+11 to 1.5E+12 sej/m^3^ (Arias and Brown, 2009; Behrend, 2007; Geber and Bjorklund, 2001; Nelson, 1998; Vassallo et al., 2009). The fact that the Cincinnati water system was the largest in size, treating an annual flow of 1.6E+08 m^3^/yr compared to 1.2E+06 to 1.2E+07 m^3^/yr for past studies, suggests that economies of scale may be a factor. Another factor that may have resulted in the lower treatment UEV is that the Cincinnati system is the only one to treat combined sewage, which likely has a lower organics concentration than sewage without stormwater and thus may be easier to treat.

**Table 2:** Emergy analysis results.

Parameter	GCWW Supply		MSDGC Sanitation	
Annual Inputs	sej/m3	sej/yr	sej/m3	sej/yr
Plant Inputs	7.8E+11	9.7E+19	6.1E+11	9.7E+19
Plant Infrastructure	8.2E+10	1.0E+19	1.2E+11	1.8E+19
Distribution/Collection Inputs	5.1E+11	6.3E+19	7.8E+10	1.2E+19
Distribution/Collection Infrastructure	4.0E+11	5.0E+19	1.0E+11	1.6E+19
Total without Distribution/Collection	8.6E+11	1.1E+20	7.3E+11	1.1E+20
Total with Distribution/Collection	1.8E+12	2.2E+20	9.1E+11	1.4E+20

**Fig. 1:**
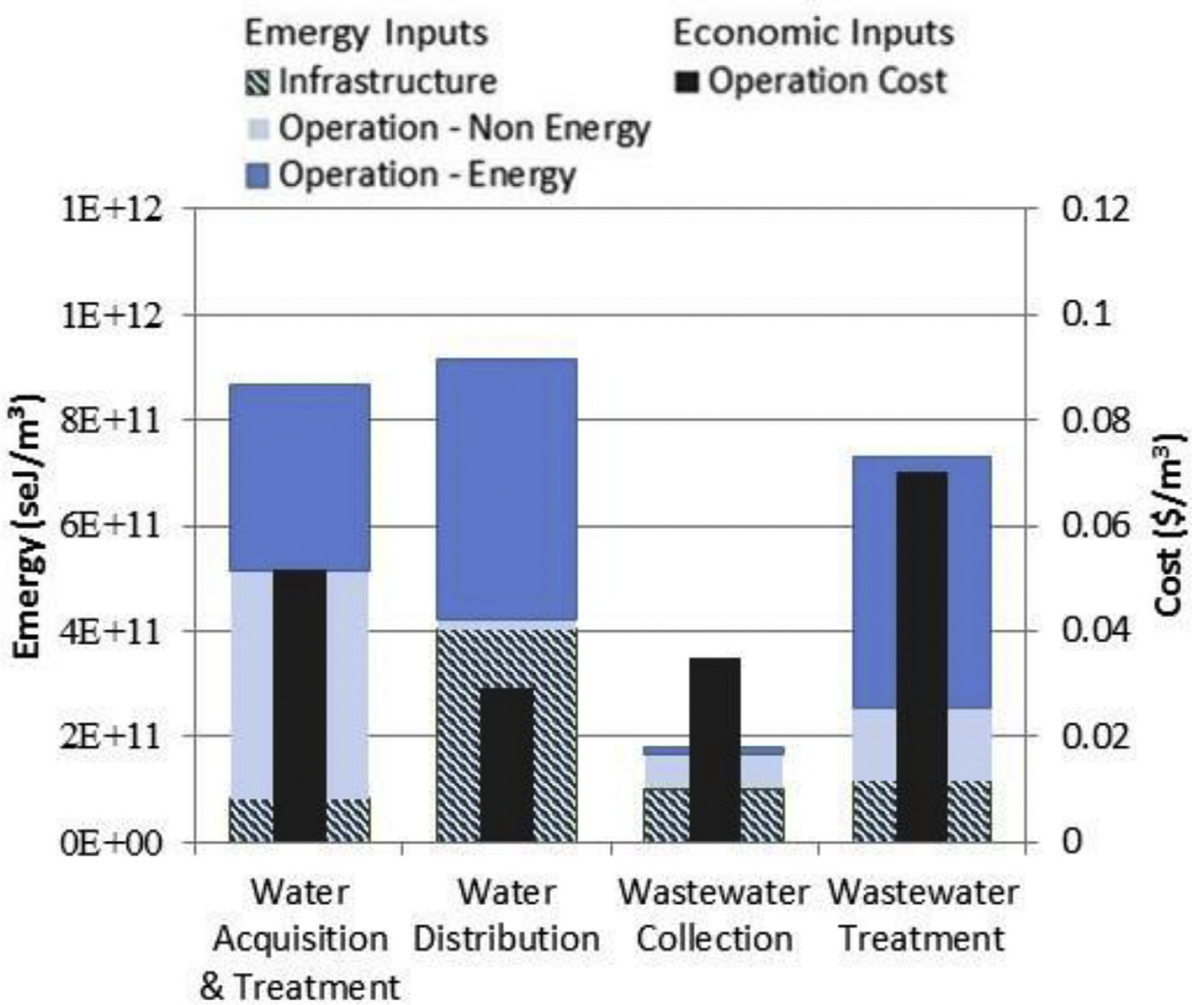
Infrastructure emergy, operation emergy and operation economic cost by major treatment stage for the greater Cincinnati urban water system.

Fig. 1 provides a breakdown of emergy inputs to the major processes in the Cincinnati UWS from source water acquisition to wastewater discharge. Each process is subdivided into emergy for infrastructure inputs, operational energy inputs (e.g. electricity, fuel, etc.) and operational non-energy inputs (e.g. labor, chemicals, etc.), and is shown alongside operational cost data.

As shown, the most resource-intensive stage is drinking water distribution, followed closely by drinking water acquisition & treatment and wastewater treatment. The high emergy inputs to the drinking water distribution system are due to the high energy inputs associated with pumping uphill owing to the location of the plant at the bottom of the Ohio River valley (and vice versa to explain the minimal energy inputs required for wastewater collection) as well as the extensive pipe network; as shown in Table 1, the total mileage of piping for the distribution system is about double that of the collection system, despite handling much less water annually. The high resource cost of drinking water treatment is in part due to the source water quality; GCWW receives its source water from the Ohio River, which is prone to contamination by upstream municipal wastewater discharge, sanitary sewer outflows and urban and agricultural storm water runoff. This is reflected both in the energy inputs required for this stage as well as the non-energy inputs, which include chemical inputs like sodium hydroxide and aluminum sulfate used for conditioning and solids adsorption, respectively. For wastewater treatment, energy inputs make up 65% of the total emergy input, mostly due to electricity required for aeration and natural gas required for sludge incineration.

**Fig. 2:**
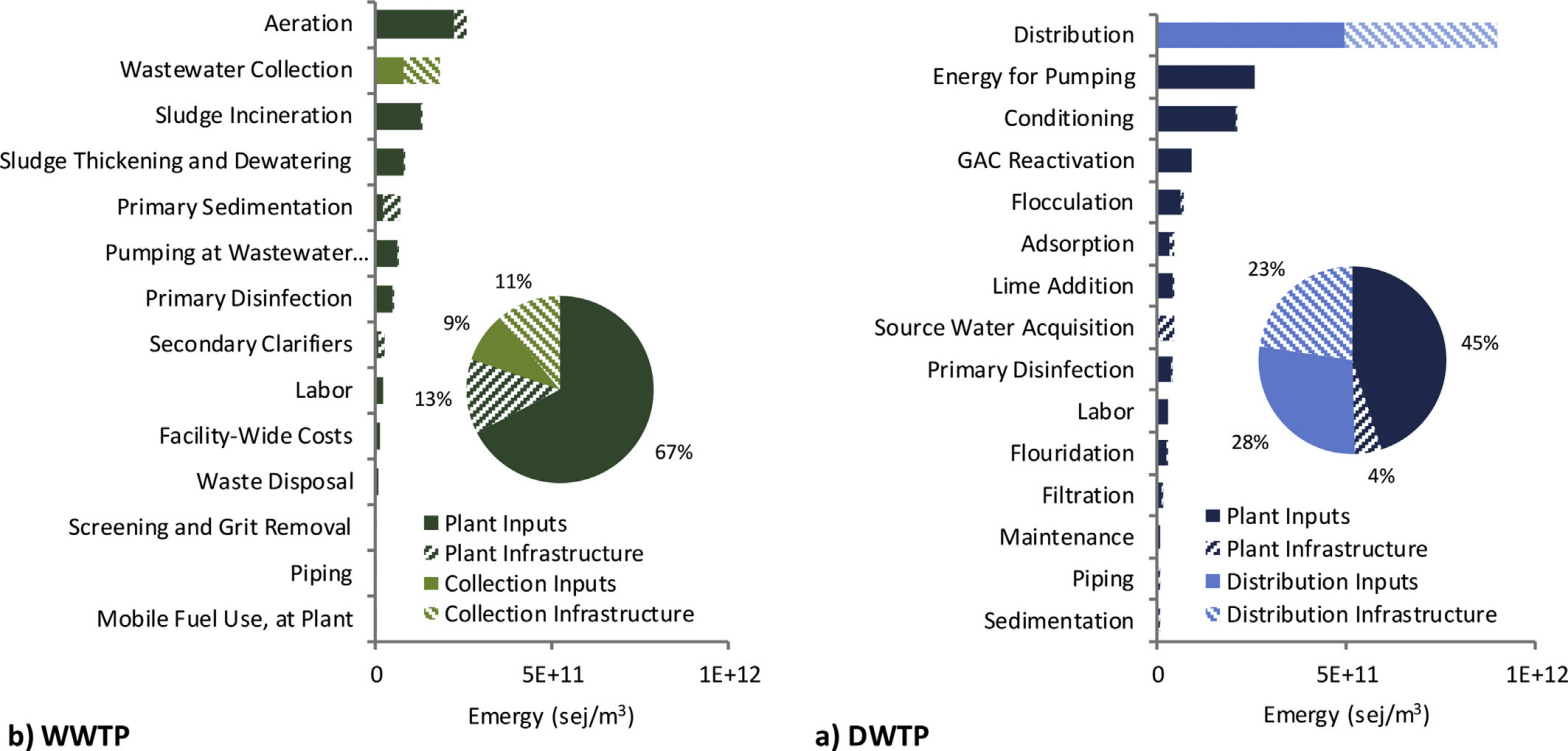
Annual and infrastructure emergy inputs to a) DWTP and b) WWTP at the plant, distribution/collection, and unit process level.

A breakdown of inputs by unit process is given in Fig. 2. In drinking water unit processes, distribution is the most resource-intensive with allocations split approximately in half between electricity for pumping and infrastructure, mostly iron piping. Following distribution are energy for pumping at the plant then conditioning with sodium hydroxide.

On the wastewater side, only 20% of the total emergy input is allocated to the collection system, while the majority of the plant inputs are allocated to the treatment process (80%). At the unit process level, aeration is the most resource intensive, all attributed to electricity. This is followed by wastewater collection then sludge incineration, which is primarily the result of natural gas use.

As the above results have shown, the total resource footprints of supply and sanitation services are largely driven by a select few unit processes, which are a function of energy, material or labor inputs. For both services, approximately 40% of the total resource footprint is attributable to electricity. For supply, this is followed by cast iron for distribution piping (20%), sodium hydroxide (11%) and natural gas (5%) which together with electricity make up 77% of the total emergy input (Table S4). On the sanitation side, electricity is followed by natural gas (14%), labor (12%) and concrete (12%) which together with electricity make up 77% of the total emergy input (Table S8). First, these rankings indicate that the total resource footprints are most sensitive to the selection of, and uncertainty in, the UEVs of these main inputs. For example, a wide range of UEVs for electricity exist in the literature for fossil-fuel based electricity. If the grid mix include sources like nuclear and wind, the uncertainty in UEV is even higher (Brown and Ulgiati, 2002; Caruso et al., 2001; Odum, 1996; Rugani et al., 2011). Second, if resource use reduction is the goal, replacing these main inputs with the ones having less emergy should be considered. For example, renewable energy sources like solar and wind generally have lower UEVs.

## 4 Discussion

### 4.1 Comparison with other metrics

The results of the LCA analysis also showed the environmental significance of energy consumption at the DWTP, WWTP and distribution system. Based on those results, it is evident that electricity for water distribution pumping, drinking water treatment in-plant pumping, and wastewater treatment aeration were the top three contributors to the environmental impact categories such as fossil fuel depletion, acidification, smog, ozone depletion, human health cancer and human health criteria. Thus, efforts to reduce energy consumption of various unit processes will be beneficial from both an emissions impact and resource appropriation perspective.

The LCA analysis did not however find comparable environmental impact associated with drinking water infrastructure, concluding that the infrastructure stage contributed less than 10% of environmental impacts with the exception of metal depletion and human noncancer impact categories. This discrepancy is due to differences in method goals and scopes. Emergy accounting takes a donor-side (or producer) perspective and captures the work done by the geobiosphere in producing a product, incorporating the time scale of material cycles. In other words, the scarcity of the resources is indirectly captured in the UEV values. LCA, on the other hand, takes a user-side (or consumer) perspective and focuses on the various environmental impacts of any product or process (Ridolfi and Bastianoni, 2008; Rugani, 2010). Emergy is therefore better able to identify use of comparably scarcer resources, providing an indication of excessive appropriation of specific resources.

In terms of operational costs, the highest are attributed to drinking water and wastewater treatment stages (Fig. 1). For drinking water, that the operational costs are greatest at the treatment plant is intuitive to a degree; ensuring the reliable production of water safe for public consumption is a complex process requiring sophisticated technology and close oversight, while the distribution phase may be relatively more ‘hands off’, largely dependent on energy for pumping, pressurized piping system and materials for extensive infrastructure networks. Interestingly, the operational emergy inputs to drinking water distribution are very high due to electricity inputs (4.92E+11 sej/m^3^, or 96% of total operational emergy) but operational costs are not, despite the largest cost input also being attributed to purchased electricity ($0.020/m^3^, or 69% of total operational cost). In contrast, the largest cost input to wastewater collection is for labor, ($0.021/m^3^, or 95% of total operational cost) though operational emergy inputs to wastewater collection are almost negligible. Thus, if utility managers were seeking to solely reduce operational costs of water supply, economic indicators may point to drinking water treatment, treatment being more costly to operate than distribution. Conversely, a focus on environmental costs (emergy) and impacts (LCA) would point towards the energy use of distribution. Vice-versa with wastewater collection, as efforts to reduce labor costs of collection would have little relative effect on treatment plant environmental burdens.

The discrepancy between cost in dollars and emergy, displayed most prominently for water distribution and wastewater collection in Fig. 1 (and Fig. S3 at the unit process level), illustrates the value of directly comparing the two accounting methods. Economic costs reflect the work done by labor in obtaining materials and energy, whereas emergy accounts for both these human services as well as the work done by the geobiosphere in generating the raw materials. For wastewater collection, the relatively minimal energy and materials reflect the resource efficiencies that can be achieved by using gravity as the source energy and large, unpressurized pipe networks to convey flows. The relatively high dollar costs, 95% of which are attributed to labor and miscellaneous operation and maintenance, are reflective of the large personnel efforts required to operate and maintain such an old conveyance system (like many historic US cities, some parts are over 100 years old). Indeed, a direct comparison of emergy to dollars at the unit process level reveals that labor has one of the greatest $/emergy ratios (Fig. S3). In comparison, the fact that unit processes such as pumping have a low $/emergy ratio may imply that the total resource costs may be underestimated if using traditional economic accounting methods.

### 4.2 Implications for future water alternatives

The large allocation of resources to the distribution system may reflect the fact that the system is designed around one quality standard (i.e. drinking water) but used for many lower quality purposes such as firefighting, irrigation, clothes washing and toilet flushing (Ma et al., 2015; Okun, 2005; Walski et al., 2001). When combined with the need to periodically flush the system to maintain adequate public health standards for both potable and non-potable purpose, these factors result in system inefficiencies and overdesign. Alternatively, drinking water systems designed around a decentralized and ‘fit for purpose’ concept such as nonpotable water reuse may be able to alleviate some of this heavy resource burden by realizing additional efficiencies (Grigg et al., 2013). Particularly in a location like Cincinnati (which is also typical of numerous other large cities located on a major river), decentralized nonpotable water reuse could reduce the degree of treatment required, which is important in a city with relatively poor quality of source water. Furthermore, decentralization holds promise for reducing the pipe network required to distribute large quantities of water.

At the WWTP, the high emergy inputs required for aeration and sludge incineration support the notion that the traditional aerobic approach to oxidize dissolved organic waste is energy and resource intensive ((NACWA) 2008). Furthermore, nutrient management requires still more resource investment to prevent eutrophication in receiving water bodies. Recent work indicates the emerging efforts to seek more comprehensive and sustainable solutions to maximize the recovery of water, energy and nutrients (Ma et al., 2015; Schoen et al., 2014; Xue et al., 2015). Biogas generation from anaerobic digestion may offset the energy consumption and address sludge production issues. Furthermore, if combined with the concept of source separation so that the nutrient and organic flows of wastewater are more concentrated, not only does a wastewater treatment plant have the possibility to be energy positive (Ma et al., 2015; McCarty et al., 2011), it can also help restore important nutrient cycles (Ma et al., 2015; Zeeman et al., 2008). Emergy could be a useful tool in weighing the additional efforts required for energy recovery, like new unit process infrastructure and labor, against the system benefits of reduced energy use, while LCA could help characterize any potential net benefits to reduced nutrient discharges.

For both plants, non-infrastructure inputs to plant treatment processes, including materials, chemicals, energy and labor are the largest emergy inputs. However, resource requirements for infrastructure are still a non-trivial component of the overall system, being 27% of the total drinking water system and 24% of the total wastewater system. This is in contrast to many urban water LCA studies which demonstrate that the contribution of infrastructure to overall impacts is small enough to justify omission of these components. This highlights an important difference between emergy, an upstream donor-side perspective which emphasizes on the total resource use including natural capital, and LCA, a downstream receiver-side perspective which focuses on the impacts of resource flows. While the downstream impacts of material usage may not be great relative to those of operational inputs, the natural capital consumption is still important, particularly for nonrenewable materials such as metals and plastics.

The comparison of economic to emergy inputs also highlights an important shortcoming of traditional economic accounting, in that appropriation of natural resources is not directly accounted for, only the services associated with acquisition and processing of the resources. When finite resources are considered in sustainability evaluations, it is imperative to couple multiple tools to better capture the complexities of water systems and provide a more complete system perspective (Xue et al., 2015).

### 4.3 A systems perspective

**Fig. 3:**
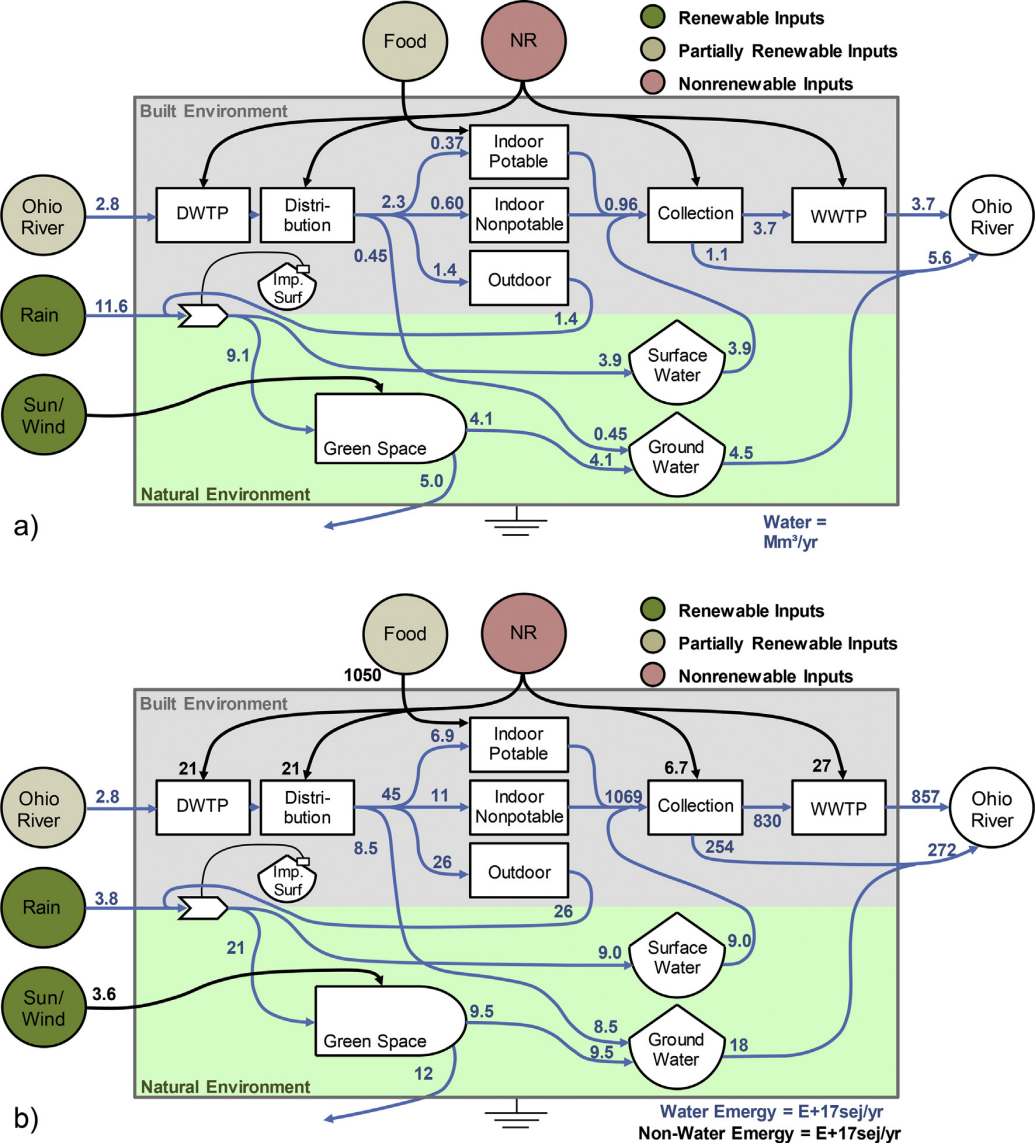
Systems Diagrams of Lick Run a) water flows and b) emergy flows.

The utility of emergy analysis is in part due to its ability to place disparate flows of material and energy on a common unit of measure, accounting for the cumulative (in space and time) resource inputs at multiple scales. At the system scale, this lends itself to evaluation of the total resource use of alternative system configurations. Fig. 3a and b shows the Lick Run UWS in terms of the major flows of water and emergy, respectively (calculations in Table S9). In these diagrams, the components of the built environment are grouped together above the components of the natural environment. This is done partially for energetic reasons (the emergy inputs to the built environment are generally more concentrated than the renewable flows supporting the natural environment, leading to greater emergy density of the built components) and partially to illustrate the fact that in its current state, the water flows within Lick Run are largely separated; water inputs to the built environment, including water treatment plants and consumers, are generally separate from water inputs to the natural environment. Only in certain cases, including leakage from distribution system and stormwater collection, do built and natural flows intersect. Greater interaction between the built and natural environment is a central theme of SUWM, IUWM, and WSUD, and as Fig. 3a illustrates great potentials for improvement in this fairly typical watershed.

Hydrologically, precipitation is the largest contributor to the system, with 11.6 million cubic meters per year (Mm^3^/yr), followed by abstraction from the Ohio River to the DWTP at 2.8 Mm^3^/yr. Of that, 13% is allocated to potable indoor use, 22% to nonpotable indoor use, 49% to nonpotable outdoor use and 16% is lost throughout the system, mostly to groundwater through pipe leaks. Of the 6.6 Mm^3^/yr of precipitation that is not evapotranspired, most becomes as groundwater recharge or stormwater runoff which, when combined with runoff from the built system (e.g. outdoor use of distributed water) results in 3.9 Mm^3^/yr of generated stormwater. Compared to the other main inputs to the collection system of potable and nonpotable indoor use (0.96 Mm^3^/yr), stormwater dominates the flow input to the wastewater system. This flow pattern is a common scenario in the U.S., as nearly 860 municipalities nationwide have combined sewer systems (CSS) (USEPA). Compared to a natural system, where 50% of rainfall is infiltrated and ultimately supports photosynthetic transpiration and healthy streamflow conditions, the flow pattern of a CSS reduces these natural processes while increasing the burden on the collection system and WWTP (U.S. Environmental Protection Agency, 2003). The implementation of green infrastructure practices throughout the watershed would restore a more natural hydrologic behavior thus promoting overall system productivity and resilience as well as reducing the burden on built infrastructure.

When viewed in terms of emergy flows, several observations become apparent. First, inputs are hierarchical, with food inputs making up the first and largest tier, followed by inputs to water infrastructure, then by renewable inputs, all separated by at least one order of magnitude. The difference between inputs of food emergy to society and inputs of nonrenewables to the built UWS clearly demonstrates the magnitude of resource inputs to the modern agricultural system and demonstrates the unique perspective offered by emergy in comparing these two system inputs. This partially explains the large expenditures in the sanitation sector, as the concentration of food-related inputs generates correspondingly large and concentrated wastewater flows which must be managed to protect human and environmental health (Kennedy et al., 2007).

Crucially, the emergy flows illustrated in Fig. 3b are overwhelmingly linear, with high-emergy water and sewage passing through the system with little to no feedback. Although the majority of the energy content of the food is extracted by human body metabolism, approximately 10% is passed in urine and feces along with important nutrients such as phosphorus, nitrogen and potassium (Rose et al., 2015). Even if only 10% of the flow in Fig. 3b could be utilized, this still represents a tremendous potential source of energy and nutrients. Under the current treatment configuration, this wastewater flow is diluted and resources have to be spent on management of these “wastes” by the WWTP. Strategies such as source separation and anaerobic digestion for energy recovery could not only reduce the treatment expenditure, but also offset upstream inputs for energy production. For example, if the system expands to the agricultural sector, the recycle pathways from the UWS (e.g. dewatered sludge, struvite, etc.) could improve the resource efficiency of food provision (Ma et al., 2015). Although such an analysis is outside the scope of the current study, the resource intensity of the current food production system and its interaction with the UWS (food-water-energy nexus) highlights the need of domestic wastewater resource recovery and incorporation of beneficial feedbacks to improve overall system efficiency.

Looking at emergy inputs other than food, nonrenewable inputs to the built environment are still an order of magnitude larger than renewable inputs to the natural environment, which represent just 2.4% of total inputs to the system (Table S12). Still, this is to be expected, as cities are not self-contained entities and require much externally (in both time and space, i.e. ancient biomass derived fossil fuel) appropriated natural capital for support. Moreover, renewable inputs to the United States in 2008 were also approximately 2% (Sweeney et al., 2007). Accordingly, the overall system sustainability can be increased by either improving the resource use efficiency of existing processes (thus lowering nonrenewable resource inputs), altering the internal configuration with other more efficient unit processes or reorganizing the internal flows of resources to create beneficial feedbacks. For example, utilizing the currently underutilized stormwater and greywater flows (mostly renewable inputs) as a nonpotable source to offset potable demand (mostly nonrenewable inputs) and WWTP load (mostly nonrenewable inputs) may represent a more sustainable and balanced system configuration. A system framework using emergy analysis allows decision makers to see the comprehensive internal interactions, calculate the degree of internal feedback relative to total inputs, identify productive vs. wasteful patterns, and holistically design urban water systems to maximize resource use efficiency.

## 5 Conclusions

This study quantified the total resource inputs to an existing UWS and placed the results within the context of the surrounding environment. In doing so, we have identified particularly resource-intensive and inefficient components of the current system allowing for recommendation of targeted improvements. Crucially, using fundamental principles of emergy theory, we suggest that the lack of internal, beneficial feedback within and between sub-systems is ultimately limiting the degree to which the competitiveness, or sustainability, of the larger system may be improved. Through the future evaluation of alternative system configurations, mainly those that incorporate internal feedbacks such as water, nutrient and/or energy reuse, we can test the hypothesis that naturally stems from this work, mainly: systems that incorporate internal, beneficial feedback mechanisms allowing for maintenance or enhancement of productivity (or level of service) at reduced levels of environmental resource appropriation will similarly reduce their level of environmental impact.

Key findings of this study include

• Centralized potable water supply, including treatment and distribution, is the most resource intensive urban water service in terms of emergy. Combined with the lack of internal feedback within the existing system, decentralized nonpotable water reuse systems could help offset potable demand, reducing the need for extensive infrastructure networks and resource-intensive potable-level treatment, particularly when source water quality (e.g. large rivers with highly developed and industrialized watersheds) is poor. Future studies should quantify the value of this feedback relative to total system inputs
• Aeration and sludge handling processes of the wastewater treatment stage that remove the organic waste fraction without utilizing any of its inherent energy are the sources of greatest impact at the wastewater treatment plant, as measured by resource use (emergy), environmental impact (LCA) and cost (LCC). Processes that obtain energy from “waste”, such as anaerobic digestion, could be used to improve the status of all three of these metrics.
• Emergy and LCA results both pointed towards drinking water treatment and drinking water distribution as environmentally burdensome stages in the urban water system, however LCA results emphasized environmental impacts associated with electricity use while emergy results emphasized energy use as well as infrastructure material demands, particularly for the distribution system. This illustrates how the two methods, used together, can substantiate the most environmentally critical aspects of a process or system, and also where using just one method may not be able to characterize the full environmental burden.
• Important insight into the sustainability of complex systems can be gained by conducting analyses that quantify resource use (emergy), environmental impacts (LCA) and cost (LCC) of the total system.

The data and framework presented here is intended to be part of an integrated sustainability framework that will be used to assess water systems for the City of Tomorrow (Ma et al., 2015). This work will eventually be combined with ongoing research in the fields of human health risk assessment, life cycle costing, life cycle assessment and resilience of UWS components to generate a truly integrated sustainability framework.

## Funding sources

This work was funded in part by the US EPA National Network for Environmental Management Studies Fellowship Program, Fellowship ID U-91755601-0.

## Disclaimer

The views expressed in this article are those of the authors and do not necessarily represent the views or policies of the U.S. Environmental Protection Agency. Mention of trade names, products, or services does not convey, and should not be interpreted as conveying, official EPA approval, endorsement or recommendation.

## Declaration of interest

The authors declare that they have no known competing financial interests or personal relationships that could have appeared to influence the work reported in this paper.

## Appendix A Supplementary data

Arden et al., 2018 Arden et al., 2018

Arden et al., 2018 SI_Tables Fig. source file Unit process diagrams, emergy tables, calculation details and data sources. This material is available free of charge via the Internet at http://pubs.acs.org Arden et al., 2018 SI_Tables Fig. source file
