# Supplementary material for: Holistic analysis of urban water systems in the Greater Cincinnati region: (2) resource use profiles by emergy accounting approach
Source: Water Res X. 2018 Nov 22;2:100012. doi: 10.1016/j.wroa.2018.100012 (PMC6415548; doi:10.1016/j.wroa.2018.100012)
Supplement: Arden et al., 2018 [file mmc1.pdf]

Supplemental Information: Holistic Analysis of Urban Water Systems in the Greater Cincinnati Region: (2) Resource Use Profiles by Emergy Accounting Approach

Sam Arden†, Xin (Cissy) Ma‡*, and Mark Brown†

†UF Center for Environmental Policy, 102 Phelps Laboratory, University of Florida, P.O. Box 116530, Gainesville, FL 32611-6350, USA

‡US EPA ORD, National Risk Management Research Laboratory, 26 West Martin Luther King Drive, Cincinnati, OH 45268, USA

* Corresponding author, ma.cissy@epa.gov

Figure S1. Unit Process Diagram of Greater Cincinnati Water Works Richard Miller Drinking Water Treatment Plant S3
Figure S2 Unit Process Diagram of Municipal Sewer District of Greater Cincinnati Mill Creek Wastewater Treatment Plant S3
Figure S3 Comparison of economic cost per volume and emergy cost per volume of major unit processes.......... S4

Table S1. GCWW DWTP annual emergy inputs S5
Table S2. GCWW DWTP plant infrastructure emergy inuts.......... S7
Table S3. GCWW distribution infrastructure emergy inputs S9
Table S4. MSD WWTP annual emergy inputs S10
Table S5. MSD WWTP plant infrastructure.......... S11
Table S6. MSD WWTP collection infrastructure emergy inputs S14
Table S7. DWTP pipe thickness for mass calculations S15
Table S8. WWTP pipe thickness for mass calculations S16
Table S9. Mass and emergy distribution among distribution system piping by size and material S17
Table S10. Mass and emergy distribution among collection system piping by size and material.......... S18
Table S11. Basin characteristics for lick run UWS emergy analysis S19
Table S12. Lick Run UWS emergy analysis S20
Table S13. UEVs.......... S24

The tables and figures provided below provide further detail for the emergy inputs to the Greater Cincinnati Water Works (GCWW) Richard Miller Drinking Water Treatment Plant (DWTP) and Municipal Sewer District of Greater Cincinnati (MSD) Mill Creek Wastewater Treatment Plant (WWTP). All emergy values in the following tables, including UEVs, have been adjusted to the 12.0E24 sej/yr global baseline (Brown et al., 2016). Tables S1-S6 show detailed inputs for the emergy analysis of the DWTP and WWTP. For each plant, there are three tables: 1) annual inputs, 2) plant infrastructure and 3) distribution system/collection system infrastructure. Each line item is normalized to the annual flow of water, 123,560,247 m^3 for the DWTP and 157,615,342 m^3 for the WWTP. For infrastructure components, materials are further normalized to an annual basis considering the lifespan of the particular input – 100 years for buildings, features and pipes, 25 years for motors and pumps (USEPA, 2014a, b). Original life cycle inventory inputs and calculations are provided in USEPA (2014a, b).

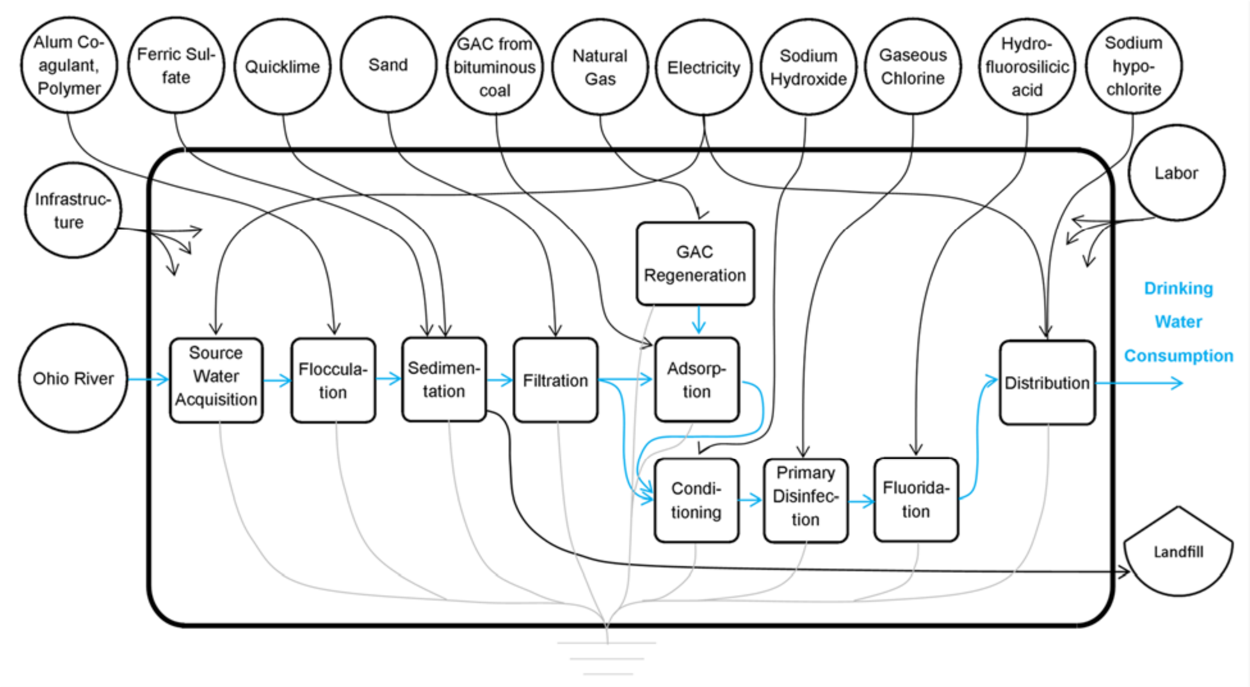

Figure S1. Unit process diagram of Greater Cincinnati Water Works Richard Miller Drinking Water Treatment Plant

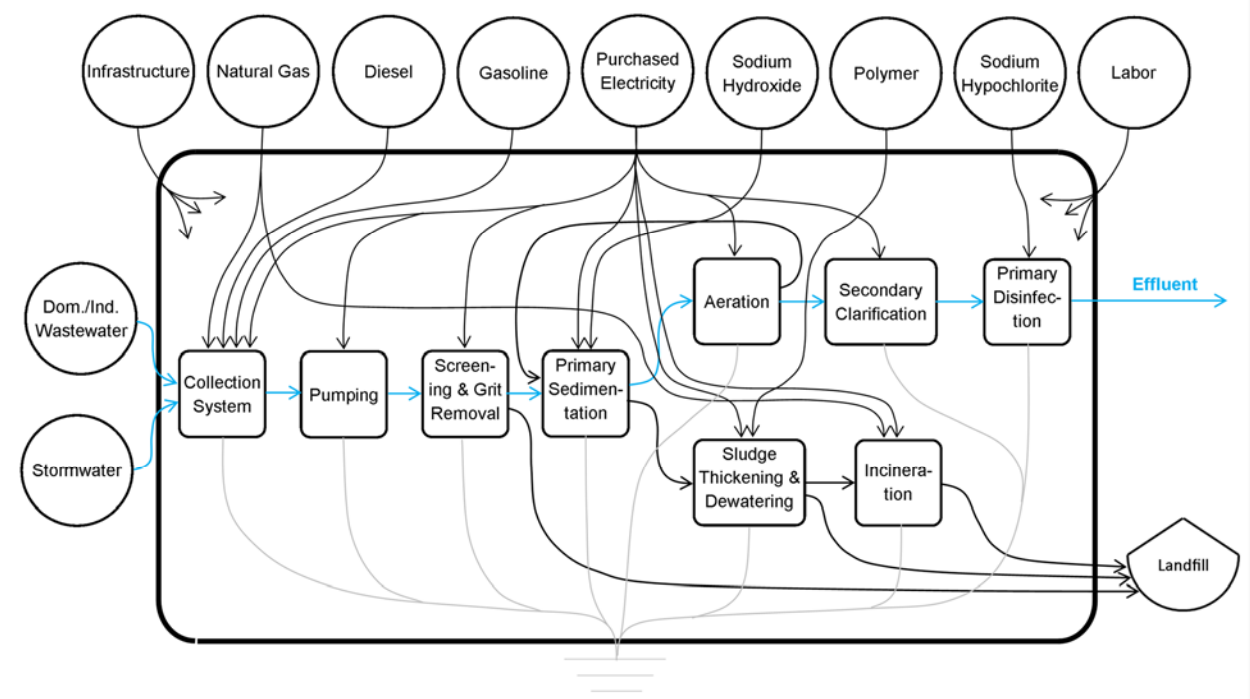

Figure S2 Unit process diagram of Municipal Sewer District of Greater Cincinnati Mill Creek Wastewater Treatment Plant

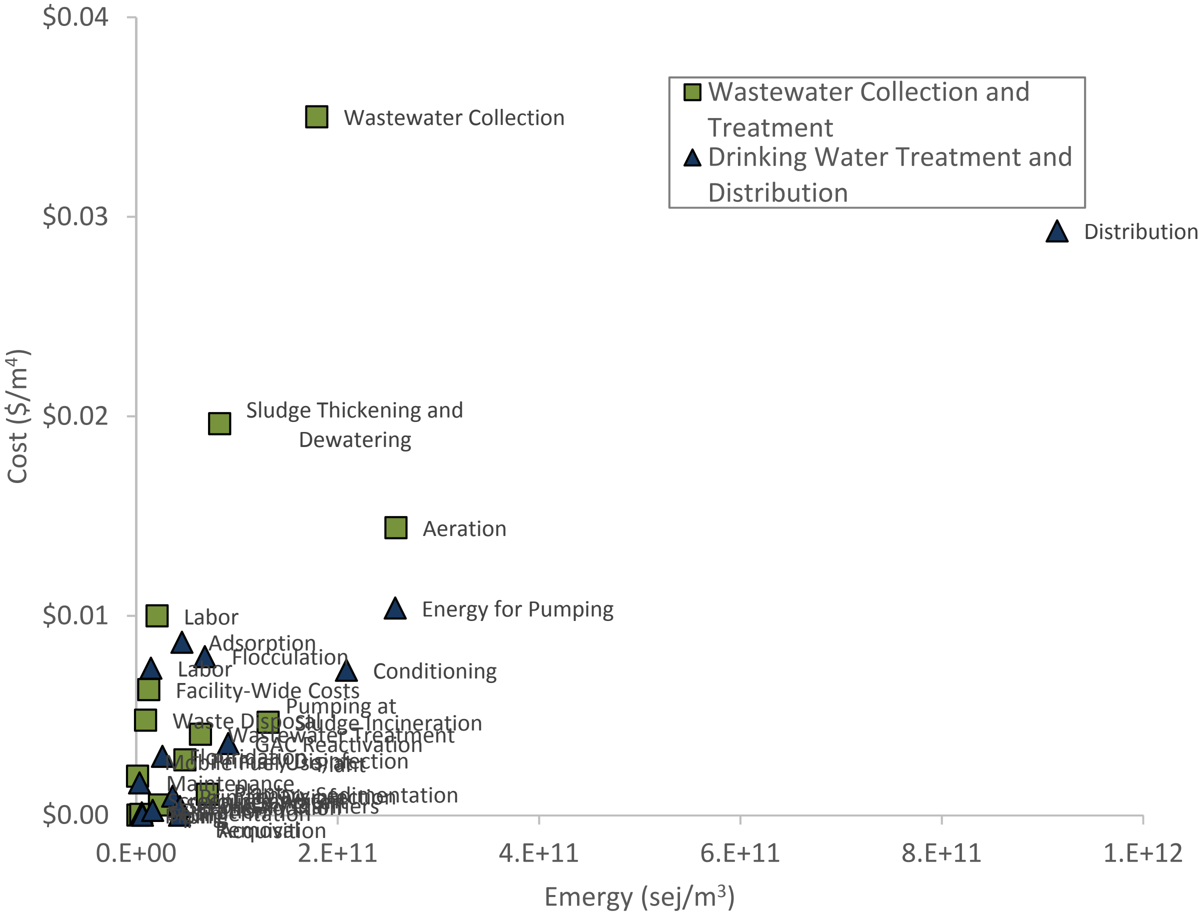

Figure S3 Comparison of economic cost per volume and emergy cost per volume of major unit processes.

Table S1. GCWW DWTP annual emergy inputs.

Item	Description	Value	Unit	UEV (sej/unit)	Emergy (sej/m³)	Dollars ($/m³)	UEV ID
Source Water Acquisition							
1	River Water	1.19	m^3	1.00E+11	1.19E+11		106
Energy for Pumping							
2	Purchased Electricity	1.14E+06	J	2.21E+05	2.52E+11	1.04E-02	119
3	Electricity from on-site hydroelectric cogeneration	6.67E+04	J	7.92E+04	5.29E+09		7
Flocculation							
4	Alum coagulant, 48% aluminum sulfate	0.019	kg	2.58E+12	5.00E+10	7.19E-03	25
5	Polymer (polyDADMAC, 10%)	0.0021	kg	4.51E+12	9.59E+09	7.61E-04	23
6	Combination truck transport, alum coagulant	0.0012	tkm	8.14E+11	9.88E+08		75
7	Combination truck transport polymer (polyDADMAC)	9.8E-04	tkm	8.14E+11	8.00E+08		75
Sedimentation							
8	Ferric sulfate	0.0014	kg	2.58E+12	3.59E+09	1.21E-04	25
9	Combination truck transport, ferric sulfate	8.6E-04	tkm	8.14E+11	7.00E+08		75
Lime Addition							
10	Quicklime at plant	0.0032	kg	1.28E+13	4.10E+10	4.37E-04	155
11	Combination truck transport, lime	1.2E-04	tkm	8.14E+11	1.01E+08		75
Filtration							
12	Sand	0.0082	kg	1.42E+12	1.16E+10	2.43E-04	36
Adsorption							
13	GAC from bituminous coal	0.0030	kg	1.09E+13	3.23E+10	8.67E-03	26
14	Combination truck transport GAC	6.5E-04	tkm	8.14E+11	5.29E+08		75
GAC Reactivation							
15	Natural gas	0.0170	m^3	5.37E+12	9.13E+10	3.59E-03	10
Conditioning							
16	Sodium hydroxide, 50%	0.027	kg	7.33E+12	1.94E+11	5.18E-03	29
17	Sodium hexametaphosphate, 30%	0.0024	kg	4.30E+12	1.04E+10	2.09E-03	28
18	Combination truck transport, sodium hydroxide	2.7E-05	tkm	8.14E+11	2.20E+07		75
19	Barge transport, sodium hydroxide	6.0E-04	tkm	9.24E+10	5.51E+07		73
20	Combination truck transport sodium hexametaphosphate	1.3E-04	tkm	8.14E+11	1.08E+08		
							75
Primary Disinfection, Gaseous Chlorine							
21	Gaseous chlorine	0.0021	kg	1.70E+13	3.56E+10	9.54E-04	27
22	Combination truck transport, gaseous chlorine	1.3E-04	tkm	8.14E+11	1.02E+08		75
23	Rail transport, gaseous chlorine	7.3E-04	tkm	2.01E+11	1.47E+08		76
Flouridation							
24	Hydrofluorosilicic acid, 24%	0.0051	kg	4.38E+12	2.22E+10	2.95E-03	30
25	Combination truck trasnport, hydrofluorosilicic acid	1.2E-04	tkm	8.14E+11	9.92E+07		75
26	Rail transport, hydrofluorosilicic acid	0.0076	tkm	2.01E+11	1.52E+09		76
Distribution							
27	Sodium hypochlorite, 15%	5.0E-04	kg	3.29E+12	1.63E+09	8.82E-05	31

Table S1. GCWW DWTP annual emergy inputs.

Item	Description	Value	Unit	UEV (sej/unit)	Emergy (sej/m³)	Dollars ($/m³)	UEV ID
28	Combination truck transport, sodium hypochlorite	2.7E-05	tkm	8.14E+11	2.20E+07		75
29	Purchased electricity	2.23E+06	J	2.21E+05	4.92E+11	2.02E-02	119
30	Facility-Wide Labor, 49 workers	7.37E-03	$	1.97E+12	1.46E+10	7.37E-03	85
31	Facility-Wide Maintenance	1.62E-03	$	1.97E+12	3.19E+09	1.62E-03	85
Labor and Maintenance							
32	Facility-Wide Labor, 49 workers	0.007	$	1.97E+12	1.46E+10	7.37E-03	85
33	Facility-Wide Maintenance	0.0016	$	1.97E+12	3.19E+09	1.62E-03	85
				**Total (with labor and service):**	**1.41E+12**	**8.09E-02**	
				**Total (without labor and service):**	**1.38E+12**		
				**Total (with labor and service, without source water):**	**1.29E+12**		

Table S2. GCWW DWTP plant infrastructure emergy inputs.

Item	Description	Value	Unit	UEV (sej/unit)	Emergy (sej/m³)	UEV ID
Source Water Acquisition						
1	Earthwork	2.84E-05	m³	9.46E+11	2.68E+07	39
2	Reinforcing Steel	5.12E-05	kg	1.43E+13	7.29E+08	55
3	6.5' Concrete Piping	2.15E-04	kg	1.37E+12	2.94E+08	44
4	Concrete	1.43E-03	kg	1.37E+12	1.97E+09	44
5	Bricks	1.87E-03	kg	2.82E+12	5.29E+09	49
6	Limestone	4.86E-03	kg	7.13E+12	3.47E+10	42
Flocculation						
7	Earthwork	7.43E-06	m³	9.46E+11	7.02E+06	39
8	Reinforcing Steel	1.15E-04	kg	1.43E+13	1.63E+09	55
9	6.5' Concrete Piping	4.80E-04	kg	1.37E+12	6.59E+08	44
10	Concrete	3.22E-03	kg	1.37E+12	4.41E+09	44
Sedimentation						
11	Earthwork	1.01E-04	m³	9.46E+11	9.57E+07	39
12	Bricks	3.70E-04	kg	2.82E+12	1.04E+09	49
Lime Addition						
13	Earthwork	7.32E-06	m³	9.46E+11	6.93E+06	39
14	Reinforcing Steel	1.43E-05	kg	1.43E+13	2.04E+08	55
15	6.5' Concrete Piping	5.99E-05	kg	1.37E+12	8.22E+07	44
16	Concrete	1.39E-03	kg	1.37E+12	1.90E+09	44
Filtration						
17	Earthwork	5.16E-06	m³	9.46E+11	4.88E+06	39
18	Reinforcing Steel	7.95E-05	kg	1.43E+13	1.13E+09	55
19	6.5' Concrete Piping	3.34E-04	kg	1.37E+12	4.58E+08	44
20	Concrete	2.23E-03	kg	1.37E+12	3.06E+09	44
Adsorption						
21	Earthwork	1.39E-05	m³	9.46E+11	1.32E+07	39
22	Reinforcing Steel	2.15E-04	kg	1.43E+13	3.06E+09	55
23	6.5' Concrete Piping	9.02E-04	kg	1.37E+12	1.24E+09	44
24	Concrete	6.02E-03	kg	1.37E+12	8.27E+09	44
Conditioning						
25	Earthwork	4.13E-06	m³	9.46E+11	3.90E+06	39
26	Reinforcing Steel	6.36E-05	kg	1.43E+13	9.07E+08	55
27	6.5' Concrete Piping	2.67E-04	kg	1.37E+12	3.66E+08	44
28	Concrete	1.78E-03	kg	1.37E+12	2.45E+09	44
Primary Disinfection						

Table S2. GCWW DWTP plant infrastructure emergy inputs.

Item	Description	Value	Unit	UEV (sej/unit)	Emergy (sej/m^3)	UEV ID
29	Earthwork	2.64E-07	m^3	9.46E+11	2.50E+05	39
30	Reinforcing Steel	4.07E-06	kg	1.43E+13	5.81E+07	55
31	6.5' Concrete Piping	1.71E-05	kg	1.37E+12	2.34E+07	44
32	Concrete	1.14E-04	kg	1.37E+12	1.56E+08	44
Flouridation						
33	Earthwork	1.23E-05	m^3	9.46E+11	1.16E+07	39
34	Concrete	1.55E-03	kg	1.37E+12	2.13E+09	44
Piping						
35	Earthwork	4.40E-06	m^3	9.46E+11	4.16E+06	39
36	Gray Iron Pipe	1.76E-04	kg	1.71E+13	3.01E+09	52
37	Ductile Iron Pipe	1.07E-04	kg	1.71E+13	1.82E+09	52
38	Concrete Pipe	9.41E-04	kg	1.37E+12	1.29E+09	44
				**Total:**	**8.25E+10**	
				**Total (sej/yr):**	**1.02E+19**	

Table S3. GCWW distribution infrastructure emergy inputs.

Item	Description	Value	Unit	UEV (sej/unit)	Emergy (sej/m³)	UEV ID
Piping						
1	Gray Iron	1.24E-02	kg	1.71E+13	2.12E+11	52
2	Ductile Iron	7.51E-03	kg	1.71E+13	1.29E+11	52
3	Concrete	2.90E-03	kg	1.37E+12	3.98E+09	44
4	Steel	1.08E-04	kg	1.88E+13	2.03E+09	53
5	Copper	3.85E-05	kg	1.53E+14	5.89E+09	50
6	PVC	3.26E-06	kg	8.38E+13	2.73E+08	66
7	HDPE	1.31E-05	kg	5.37E+12	7.06E+07	125
8	Transite	1.30E-04	kg	2.63E+12	3.41E+08	51
Water Storage Tanks and Reservoirs						
9	Concrete	7.69E-05	kg	1.37E+12	1.06E+08	44
10	Steel	6.45E-05	kg	1.88E+13	1.21E+09	53
11	Earthwork	2.88E-05	m³	9.46E+11	2.72E+07	39
Motors, Pumps and Valves						
12	Steel	0.0021	kg	1.88E+13	3.97E+10	53
13	Electrical Steel	4.1E-05	kg	1.27E+14	5.16E+09	56
14	Stainless 18/8 coil	4.5E-06	kg	1.31E+14	5.86E+08	58
15	Cast Iron	9.9E-05	kg	1.71E+13	1.70E+09	52
16	Aluminum	2.4E-06	kg	8.19E+13	1.95E+08	59
17	Copper	7.1E-06	kg	1.53E+14	1.08E+09	50
				**Total:**	**4.03E+11**	
				**Total (sej/yr):**	**4.98E+19**	

Table S4. MSD WWTP annual emergy inputs.

Item	Description	Value	Unit	UEV (sej/unit)	Emergy (sej/m³)	Energy (J'/m³)	Dollars ($/m³)	UEV ID
Wastewater Collection								
1	Stormwater	0.24	m³	3.65E+11	8.82E+10			
2	Industrial and Household Water	1.00	m³	1.71E+13	1.71E+13			
3	Purchased Electricity	0.0067	kWh	7.95E+11	5.30E+09	2.4E+04	3.47E-04	109
4	Natural Gas	3.4E-04	m³	5.37E+12	1.83E+09	1.3E+04	6.40E-05	10
5	Diesel	7.8E-07	m³	5.12E+09	3.97E+03	2.8E+04		16
6	Gasoline	1.17E-06	m³	4.74E+15	5.56E+09	3.8E+04	1.26E-03	19
7	Labor	0.021	$	1.97E+12	4.14E+10		2.10E-02	85
8	Other O&M	0.012	$	1.97E+12	2.43E+10		1.23E-02	85
Pumping at Wastewater Treatment Plant								
9	Purchased Electricity	0.078	kWh	7.95E+11	6.18E+10	2.8E+05	4.05E-03	109
Screening and Grit Removal								
10	Purchased Electricity	6.2E-04	kWh	7.95E+11	4.91E+08	2.2E+03	3.22E-05	109
Primary Sedimentation								
11	Purchased Electricity	0.0086	kWh	7.95E+11	6.82E+09	3.1E+04	4.47E-04	109
12	Sodium Hydroxide	0.0020	kg	7.33E+12	1.44E+10		6.00E-04	29
Sludge Thickening and Dewatering								
13	Purchased Electricity	0.060	kWh	7.95E+11	4.77E+10	2.2E+05	3.12E-03	109
14	Polymer (polyacrylamide)	0.0069	kg	4.51E+12	3.10E+10		1.65E-02	23
Sludge Incineration								
15	Purchased Electricity	0.0066	kWh	7.95E+11	5.24E+09	2.4E+04	3.43E-04	109
16	Natural Gas	0.023	m³	5.37E+12	1.25E+11	8.9E+05	4.31E-03	10
Aeration								
17	Purchased Electricity	0.28	kWh	7.95E+11	2.20E+11	9.9E+05	1.44E-02	109
Secondary Clarifiers								
18	Purchased Electricity	0.0097	kWh	7.95E+11	7.75E+09	3.5E+04	5.08E-04	109
Primary Disinfection								
19	Sodium hypochlorite	0.012	liters	3.95E+12	4.85E+10		2.77E-03	154
Mobile Fuel Combustion, at Plant								
20	Diesel	1.01E-06	m³	5.12E+09	5.19E+03	3.3E+04	1.52E-03	16
21	Gasoline	3.1E-07	m³	4.74E+15	1.46E+09	1.1E+04	4.23E-04	19
Facility-Wide Costs								
22	Materials - Unspecified	6.40E-03	$	1.97E+12	1.26E+10		6.29E-03	85
23	Labor	9.18E-03	$	1.97E+12	1.81E+10		8.78E-03	85
24	Service	1.35E-03	$	1.97E+12	2.67E+09		1.20E-03	85
25	Waste Disposal	4.76E-03	$	1.97E+12	9.39E+09		4.76E-03	85
				**Total (with labor and service):**	**1.79E+13**		**1.05E-01**	
				**Total (without labor and service):**	**1.78E+13**			
				**Total (with labor and service, without source water):**	**6.91E+11**			

Table S5. MSD WWTP plant infrastructure.

Item	Description	Value	Unit	UEV (sej/unit)	Emergy (sej/m³)	UEV ID
Pumping at Wastewater Treatment Plant						
1	Concrete	1.61E-05	kg	1.37E+12	2.21E+07	44
2	Steel	3.90E-05	kg	1.88E+13	7.34E+08	53
3	Cast Iron	2.76E-05	kg	1.71E+13	4.72E+08	52
4	Stainless Steel 18/8 Coil	2.20E-06	kg	1.31E+14	2.88E+08	57
5	Electrical Steel	3.70E-06	kg	1.27E+14	4.70E+08	56
6	Other Steel	7.90E-07	kg	1.88E+13	1.49E+07	53
7	Aluminum	2.10E-07	kg	8.19E+13	1.72E+07	59
8	Copper	6.40E-07	kg	1.53E+14	9.79E+07	50
9	HDPE	0.00E+00	kg	5.37E+12	0.00E+00	125
10	Earthwork	2.60E-06	m^3	9.46E+11	2.46E+06	39
Screening and Grit Removal						
11	Concrete	1.63E-03	kg	1.37E+12	2.24E+09	44
12	Steel	9.80E-05	kg	1.88E+13	1.84E+09	53
13	Cast Iron	7.30E-08	kg	1.71E+13	1.25E+06	52
14	Stainless Steel 18/8 Coil	8.40E-09	kg	1.31E+14	1.10E+06	57
15	Electrical Steel	1.30E-08	kg	1.27E+14	1.65E+06	56
16	Other Steel	3.60E-09	kg	1.88E+13	6.77E+04	53
17	Aluminum	3.90E-09	kg	8.19E+13	3.19E+05	59
18	Copper	2.80E-09	kg	1.53E+14	4.28E+05	50
19	HDPE	1.20E-06	kg	5.37E+12	6.44E+06	125
20	Earthwork	2.60E-06	m^3	9.46E+11	2.46E+06	39
Primary Sedimentation						
21	Concrete	2.40E-02	kg	1.37E+12	3.29E+10	44
22	Steel	8.80E-04	kg	1.88E+13	1.66E+10	53
23	Cast Iron	3.10E-07	kg	1.71E+13	5.30E+06	52
24	Stainless Steel 18/8 Coil	9.10E-08	kg	1.31E+14	1.19E+07	57
25	Electrical Steel	2.40E-08	kg	1.27E+14	3.05E+06	56
26	Other Steel	6.00E-09	kg	1.88E+13	1.13E+05	53
27	Aluminum	5.40E-09	kg	8.19E+13	4.42E+05	59
28	Copper	4.60E-09	kg	1.53E+14	7.03E+05	50
29	HDPE	5.80E-06	kg	5.37E+12	3.11E+07	125
30	Earthwork	6.10E-07	m^3	9.46E+11	5.77E+05	39
Aeration						
31	Concrete	1.75E-02	kg	1.37E+12	2.40E+10	44
32	Steel	6.40E-04	kg	1.88E+13	1.20E+10	53

Table S5. MSD WWTP plant infrastructure.

Item	Description	Value	Unit	UEV (sej/unit)	Emergy (sej/m³)	UEV ID
33	Cast Iron	1.30E-05	kg	1.71E+13	2.22E+08	52
34	Electrical Steel	1.30E-05	kg	1.27E+14	1.65E+09	56
35	Other Steel	2.80E-06	kg	1.88E+13	5.27E+07	53
36	Aluminum	7.60E-07	kg	8.19E+13	6.22E+07	59
37	Copper	2.30E-06	kg	1.53E+14	3.52E+08	50
38	Earthwork	2.40E-06	m³	9.46E+11	2.27E+06	39
Secondary Clarifiers						
39	Concrete	7.44E-03	kg	1.37E+12	1.02E+10	44
40	Steel	2.70E-04	kg	1.88E+13	5.08E+09	53
41	Cast Iron	4.92E-06	kg	1.71E+13	8.42E+07	52
42	Stainless Steel 18/8 Coil	7.20E-07	kg	1.31E+14	9.42E+07	57
43	Electrical Steel	5.10E-08	kg	1.27E+14	6.48E+06	56
44	Other Steel	1.40E-08	kg	1.88E+13	2.63E+05	53
45	Aluminum	1.40E-08	kg	8.19E+13	1.15E+06	59
46	Copper	1.10E-08	kg	1.53E+14	1.68E+06	50
47	Earthwork	1.50E-06	m³	9.46E+11	1.42E+06	39
Sludge Thickening and Dewatering						
48	Concrete	1.13E-03	kg	1.37E+12	1.55E+09	44
49	Steel	1.20E-04	kg	1.88E+13	2.26E+09	53
50	Cast Iron	2.88E-06	kg	1.71E+13	4.93E+07	52
51	Stainless Steel 18/8 Coil	1.90E-07	kg	1.31E+14	2.49E+07	57
52	Electrical Steel	2.40E-06	kg	1.27E+14	3.05E+08	56
53	Other Steel	5.20E-07	kg	1.88E+13	9.78E+06	53
54	Aluminum	1.60E-07	kg	8.19E+13	1.31E+07	59
55	Copper	4.20E-07	kg	1.53E+14	6.42E+07	50
56	HDPE	1.60E-05	kg	5.37E+12	8.59E+07	125
57	Earthwork	4.90E-06	m³	9.46E+11	4.64E+06	39
Sludge Incineration						
58	Concrete	1.22E-05	kg	1.37E+12	1.68E+07	44
59	Steel	5.80E-05	kg	1.88E+13	1.09E+09	53
60	Cast Iron	4.00E-07	kg	1.71E+13	6.84E+06	52
61	Stainless Steel 18/8 Coil	5.70E-08	kg	1.31E+14	7.46E+06	57
62	Electrical Steel	9.50E-08	kg	1.27E+14	1.21E+07	56
63	Other Steel	2.10E-08	kg	1.88E+13	3.95E+05	53
64	Aluminum	6.40E-09	kg	8.19E+13	5.24E+05	59

Table S5. MSD WWTP plant infrastructure.

Item	Description	Value	Unit	UEV (sej/unit)	Emergy (sej/m³)	UEV ID
65	Copper	1.70E-08	kg	1.53E+14	2.60E+06	50
66	Earthwork	1.90E-06	m³	9.46E+11	1.80E+06	39
Primary Disinfection						
67	HDPE	2.20E-06	kg	5.37E+12	1.18E+07	125
Piping †						
68	Ductile Iron	3.09E-05	kg	1.71E+13	5.28E+08	52
69	Reinforced Concrete	9.22E-04	kg	1.37E+12	1.26E+09	44
70	Earthwork	3.52E-06	m³	9.46E+11	3.33E+06	39
				**Total:**	**1.17E+11**	
				**Total (sej/yr):**	**1.84E+19**	

Table S6. MSD collection infrastructure emergy inputs.

Item	Description	Value	Unit	UEV (sej/unit)	Emergy (sej/m³)	UEV ID
Piping †						
1	PVC	5.47E-04	kg	8.38E+13	4.59E+10	66
2	Vitrified Clay	5.58E-03	kg	2.82E+12	1.57E+10	49
3	Concrete	1.31E-02	kg	1.37E+12	1.80E+10	44
4	Reinforced Concrete	1.24E-02	kg	1.37E+12	1.70E+10	44
5	Cement-Lined Ductile Iron - Ductile Iron	2.28E-04	kg	1.71E+13	3.90E+09	52
6	Cement-Lined Ductile Iron - Cement	2.72E-05	kg	2.75E+12	7.48E+07	37
7	Earthwork	3.85E-04	m^3	9.46E+11	3.64E+08	39
				**Total:**	**1.01E+11**	
				**Total (sej/yr):**	**1.59E+19**	

Table S7. DWTP pipe thickness for mass calculations

Nominal Pipe Size (in)	Wall Thickness (m)							
	Gray Iron[1]	Ductile Iron[1]	Concrete[2]	Steel[3]	Copper[4,5]	PVC[6,7]	HDPE[8]	Transite[9]
0.75	6.35E-03	6.35E-03	5.00E-02	6.35E-03	1.14E-03	4.88E-03	2.97E-03	5.00E-02
1.00	6.35E-03	6.35E-03	5.00E-02	6.35E-03	1.27E-03	4.88E-03	3.71E-03	5.00E-02
1.50	6.35E-03	6.35E-03	5.00E-02	6.35E-03	1.52E-03	4.88E-03	5.36E-03	5.00E-02
2.00	6.35E-03	6.35E-03	5.00E-02	6.35E-03	1.78E-03	4.88E-03	6.71E-03	5.00E-02
2.50	6.35E-03	6.35E-03	5.00E-02	6.35E-03	2.03E-03	4.88E-03	6.63E-03	5.00E-02
3	6.35E-03	6.35E-03	5.00E-02	6.35E-03	2.29E-03	4.88E-03	8.08E-03	5.00E-02
4	6.35E-03	6.35E-03	5.00E-02	6.35E-03	2.79E-03	6.78E-03	8.46E-03	5.00E-02
6	6.35E-03	6.35E-03	5.00E-02	6.35E-03	3.56E-03	9.73E-03	1.25E-02	5.00E-02
8	6.35E-03	6.35E-03	5.00E-02	6.35E-03	5.08E-03	1.28E-02	1.62E-02	5.00E-02
10	6.61E-03	6.61E-03	5.00E-02	6.61E-03	6.35E-03	1.57E-02	2.02E-02	5.00E-02
12	7.11E-03	7.11E-03	5.10E-02	7.11E-03	7.11E-03	1.86E-02	2.40E-02	5.10E-02
16	8.13E-03	8.13E-03	5.40E-02	8.13E-03	9.36E-03	1.56E-02	3.01E-02	5.40E-02
20	9.15E-03	9.15E-03	6.00E-02	9.15E-03	1.15E-02	1.95E-02	3.76E-02	6.00E-02
24	1.02E-02	1.02E-02	6.40E-02	1.02E-02	1.37E-02	2.34E-02	4.52E-02	6.40E-02
30	1.07E-02	1.07E-02	7.00E-02	1.07E-02	1.70E-02	2.93E-02	5.64E-02	7.00E-02
35	1.19E-02	1.19E-02	7.60E-02	1.19E-02	1.97E-02	3.52E-02	5.38E-02	7.60E-02
36	1.19E-02	1.19E-02	7.60E-02	1.19E-02	2.02E-02	3.52E-02	5.38E-02	7.60E-02
42	1.32E-02	1.32E-02	8.90E-02	1.32E-02	2.35E-02	4.25E-02	5.08E-02	8.90E-02
44	1.37E-02	1.37E-02	9.33E-02	1.37E-02	2.46E-02	4.45E-02	2.81E-02	9.33E-02
46	1.42E-02	1.42E-02	9.77E-02	1.42E-02	2.57E-02	4.65E-02	5.81E-02	9.77E-02
48	1.47E-02	1.47E-02	1.02E-01	1.47E-02	2.67E-02	4.85E-02	5.81E-02	1.02E-01
54	1.65E-02	1.65E-02	1.14E-01	1.65E-02	3.00E-02	5.45E-02	6.53E-02	1.14E-01
60	1.73E-02	1.73E-02	1.27E-01	1.73E-02	3.33E-02	6.05E-02	6.16E-02	1.27E-01
72	2.00E-02	2.00E-02	2.00E-01	--	--	--	--	2.00E-01

[1]ANSI/AWWA C151/A21.51-02 Ductile Iron Pipe, Centrifigually Cast, For Water - assume anything less than 3 inches is .25 in thick.
[2]AWWA C302-11 Reinforced Concrete Pressure Pipe, Noncylinder Type.
[3]ANSI/AWWA C151/A21.51-02 Ductile Iron Pipe, Centrifigually Cast, For Water - assume anything less than 3 inches is .25 in thick.
[4]ASTM B88, assume type L.
[5]16" and larger derived from linear regression of smaller Size/Thickness relationships.
[6]AWWA C900-07 Polyvinyl Chloride (PVC) Pressure Pipe and Fabricated Fittings, 4 In. Through 12 In., for Water Transmission and Distribution.
[7]AWWA C905-10 Polyvinyl Chloride (PVC) Pressure Pipe and Fabricated Fittings, 14 In. Through 48 In., for Water Transmission and Distribution.
[8]ASTM F2619, assume middle DR rating.
[9]ASTM C296.

Table S8. WWTP pipe thickness for mass calculations

	Wall Thickness (m)					
Nominal Pipe Size (in)	PVC[1]	Vitrified Clay[2]	Concrete[3]	Reinforced Concrete[4]	Cement-Lined Duct. Iron[5]	Cement for Lining[6]
8	8.43E-03	2.15E-02	5.08E-02	5.08E-02	6.35E-03	1.59E-03
10	1.05E-02	2.64E-02	5.08E-02	5.08E-02	6.61E-03	1.59E-03
12	1.24E-02	3.19E-02	5.08E-02	5.08E-02	7.11E-03	1.59E-03
15	1.52E-02	3.77E-02	5.72E-02	5.72E-02	7.62E-03	2.38E-03
16	1.56E-02	5.17E-02	6.35E-02	6.35E-02	7.62E-03	2.38E-03
18	1.76E-02	5.17E-02	6.35E-02	6.35E-02	7.88E-03	2.38E-03
20	1.95E-02	5.74E-02	6.99E-02	6.99E-02	8.38E-03	2.38E-03
21	2.35E-02	5.74E-02	6.99E-02	6.99E-02	9.40E-03	2.38E-03
24	2.35E-02	6.33E-02	7.62E-02	7.62E-02	9.40E-03	2.38E-03
27	2.93E-02	7.24E-02	8.26E-02	8.26E-02	1.07E-02	3.18E-03
30	2.93E-02	8.04E-02	8.89E-02	8.89E-02	1.07E-02	3.18E-03
33	3.52E-02	8.83E-02	9.53E-02	9.53E-02	1.19E-02	3.18E-03
36	3.52E-02	9.63E-02	1.02E-01	1.02E-01	1.19E-02	3.18E-03
42	--	1.12E-01	1.14E-01	1.14E-01	--	--
48	--	--	1.27E-01	1.27E-01	--	--
54	--	--	1.40E-01	1.40E-01	--	--
60	--	--	1.52E-01	1.52E-01	--	--
66	--	--	1.65E-01	1.65E-01	--	--
72	--	--	1.78E-01	1.78E-01	--	--
96	--	--	2.29E-01	2.29E-01	--	--

[1] ASTM D2241, assumed SDR26.
[2] http://www.loganclaypipe.com/pp_pipespecsdata.html
[3] same as RCP, verified at http://www.mcphersonconcrete.com/pipe-specs.htm
[4] ASTM C76, Assumed Class III Wall B.
[5] ASTM A746.
[6] ANSI C104 Cement Lining.

Table S9. Mass and emergy distribution among distribution system piping by size and material

	Density of Material (kg/m³)							
Distribution	Gray Iron	Ductile Iron	Concrete	Steel	Copper	PVC	HDPE	Transite
Infrastructure	7100	7100	2400	7850	8940	1400	950	1750
Nominal Pipe	Mass per Volume of Water per Year (kg/(m³yr))							
Size (inches)	Gray Iron	Ductile Iron	Concrete	Steel	Copper	PVC	HDPE	Transite
0.75	8.29E-09	5.02E-09	5.40E-09	7.21E-11	3.10E-12	6.47E-13	1.06E-12	2.41E-10
1	3.04E-08	1.84E-08	1.73E-08	2.65E-10	1.34E-11	2.40E-12	5.13E-12	7.74E-10
1.5	6.66E-08	4.04E-08	3.16E-08	5.80E-10	3.73E-11	5.34E-12	1.73E-11	1.41E-09
2	2.35E-06	1.42E-06	9.93E-07	2.05E-08	1.58E-09	1.90E-10	7.86E-10	4.44E-08
3	2.20E-07	1.33E-07	8.05E-08	1.91E-09	1.97E-10	1.79E-11	8.97E-11	3.60E-09
4	2.32E-05	1.41E-05	7.81E-06	2.02E-07	2.58E-08	2.68E-09	9.92E-09	3.49E-07
6	1.69E-03	1.02E-03	5.17E-04	1.47E-05	2.43E-06	2.85E-07	1.08E-06	2.31E-05
8	2.76E-03	1.67E-03	7.98E-04	2.40E-05	5.73E-06	6.16E-07	2.32E-06	3.57E-05
10	1.56E-04	9.42E-05	4.18E-05	1.35E-06	3.91E-07	4.12E-08	1.58E-07	1.87E-06
12	1.59E-03	9.63E-04	3.96E-04	1.38E-05	4.16E-06	4.65E-07	1.78E-06	1.77E-05
16	2.78E-04	1.69E-04	4.98E-05	2.42E-06	8.36E-07	5.66E-08	3.06E-07	2.23E-06
20	8.25E-04	4.99E-04	1.48E-04	7.18E-06	2.71E-06	1.86E-07	1.01E-06	6.61E-06
24	6.40E-04	3.87E-04	1.12E-04	5.57E-06	2.24E-06	1.56E-07	8.42E-07	4.99E-06
30	1.78E-04	1.08E-04	3.28E-05	1.55E-06	7.35E-07	5.15E-08	2.78E-07	1.47E-06
35	9.29E-04	5.63E-04	1.67E-04	8.09E-06	3.97E-06	2.87E-07	1.25E-06	7.46E-06
36	1.09E-03	6.63E-04	1.97E-04	9.52E-06	4.80E-06	3.38E-07	1.48E-06	8.80E-06
42	2.88E-04	1.74E-04	5.47E-05	2.50E-06	1.32E-06	9.70E-08	3.36E-07	2.45E-06
44	9.98E-04	6.04E-04	1.92E-04	8.68E-06	4.63E-06	3.39E-07	1.17E-06	8.57E-06
46	1.31E-04	7.93E-05	2.54E-05	1.14E-06	6.11E-07	4.49E-08	1.55E-07	1.13E-06
48	5.12E-04	3.10E-04	1.00E-04	4.45E-06	2.40E-06	1.76E-07	6.11E-07	4.47E-06
54	1.36E-04	8.26E-05	2.66E-05	1.19E-06	6.42E-07	4.72E-08	1.64E-07	1.19E-06
60	1.78E-04	1.08E-04	3.69E-05	1.55E-06	8.85E-07	6.52E-08	1.93E-07	1.65E-06
**Total Mass:**	**1.24E-02**	**7.51E-03**	**2.90E-03**	**1.08E-04**	**3.85E-05**	**3.26E-06**	**1.31E-05**	**1.30E-04**
**Proportion:**	**54%**	**33%**	**13%**	**0%**	**0%**	**0%**	**0%**	**1%**

	UEV of Material (sej/kg)							
Distribution	Gray Iron	Ductile Iron	Concrete	Steel	Copper	PVC	HDPE	Transite
Infrastructure	1.71E+13	1.71E+13	1.37E+12	1.88E+13	7.43E+13	8.14E+13	8.10E+12	2.63E+12
Nominal Pipe	Emergy per Volume of Water per Year (sej/(m³yr))							
Size (inches)	Gray Iron	Ductile Iron	Concrete	Steel	Copper	PVC	HDPE	Transite
0.75	1.42E+05	8.59E+04	7.41E+03	1.36E+03	2.30E+02	5.26E+01	8.56E+00	6.35E+02
1	5.21E+05	3.15E+05	2.38E+04	4.98E+03	9.93E+02	1.96E+02	4.15E+01	2.04E+03
1.5	1.14E+06	6.90E+05	4.34E+04	1.09E+04	2.77E+03	4.34E+02	1.40E+02	3.72E+03
2	4.02E+07	2.43E+07	1.36E+06	3.85E+05	1.18E+05	1.54E+04	6.36E+03	1.17E+05
3	3.76E+06	2.28E+06	1.10E+05	3.60E+04	1.46E+04	1.45E+03	7.27E+02	9.47E+03
4	3.97E+08	2.41E+08	1.07E+07	3.80E+06	1.92E+06	2.18E+05	8.03E+04	9.19E+05
6	2.89E+10	1.75E+10	7.09E+08	2.77E+08	1.81E+08	2.32E+07	8.78E+06	6.08E+07
8	4.71E+10	2.86E+10	1.09E+09	4.51E+08	4.26E+08	5.01E+07	1.88E+07	9.39E+07
10	2.66E+09	1.61E+09	5.74E+07	2.55E+07	2.90E+07	3.35E+06	1.28E+06	4.92E+06
12	2.72E+10	1.65E+10	5.43E+08	2.60E+08	3.09E+08	3.78E+07	1.44E+07	4.66E+07
16	4.76E+09	2.88E+09	6.83E+07	4.56E+07	6.21E+07	4.60E+06	2.48E+06	5.86E+06
20	1.41E+10	8.54E+09	2.03E+08	1.35E+08	2.01E+08	1.51E+07	8.15E+06	1.74E+07
24	1.09E+10	6.63E+09	1.53E+08	1.05E+08	1.67E+08	1.27E+07	6.82E+06	1.31E+07
30	3.05E+09	1.85E+09	4.50E+07	2.92E+07	5.46E+07	4.19E+06	2.25E+06	3.86E+06
35	1.59E+10	9.63E+09	2.29E+08	1.52E+08	2.95E+08	2.34E+07	1.02E+07	1.96E+07
36	1.87E+10	1.13E+10	2.70E+08	1.79E+08	3.57E+08	2.75E+07	1.20E+07	2.32E+07
42	4.92E+09	2.98E+09	7.51E+07	4.71E+07	9.84E+07	7.89E+06	2.72E+06	6.44E+06
44	1.71E+10	1.03E+10	2.63E+08	1.63E+08	3.44E+08	2.76E+07	9.50E+06	2.25E+07
46	2.24E+09	1.36E+09	3.48E+07	2.14E+07	4.54E+07	3.65E+06	1.26E+06	2.99E+06
48	8.75E+09	5.30E+09	1.37E+08	8.38E+07	1.79E+08	1.44E+07	4.95E+06	1.18E+07
54	2.33E+09	1.41E+09	3.65E+07	2.23E+07	4.77E+07	3.84E+06	1.32E+06	3.13E+06
60	3.04E+09	1.84E+09	5.06E+07	2.91E+07	6.58E+07	5.30E+06	1.57E+06	4.34E+06
**Total Emergy:**	**2.12E+11**	**1.29E+11**	**3.98E+09**	**2.03E+09**	**2.86E+09**	**2.65E+08**	**1.06E+08**	**3.41E+08**
**Proportion:**	**61%**	**37%**	**1%**	**1%**	**1%**	**0%**	**0%**	**0%**

Note: Green and red shading indicate relative magnitude of emergy distributions; green shading is a comparison by size and material, red shading is a comparison of material totals.

Table S10. Mass and emergy distribution among collection system piping by size and material

Collection System	PVC	Vitrified Clay	Concrete	Reinforced Concrete	Cement-Lined Ductile Iron	Cement for Lining
	Density of Material (kg/m^3)					
	1400	2245	2400	2400	7100	3150
	Mass per Volume of Water per Year ($kg/(m^3yr)$)					
**Nominal Pipe Size (in)**	**PVC**	**Vitrified Clay**	**Concrete**	**Reinforced Concrete**	**Cement-Lined Ductile Iron**	**Cement for Lining**
8	1.39E-04	8.17E-04	8.63E-04	2.29E-05	1.60E-05	1.73E-06
10	4.38E-07	1.31E-04	5.37E-05	2.18E-06	1.22E-06	1.27E-07
12	8.09E-05	1.96E-03	6.22E-03	5.22E-04	3.41E-05	3.32E-06
15	2.68E-05	5.78E-04	4.08E-04	7.93E-05	0.00E+00	0.00E+00
16	0.00E+00	0.00E+00	0.00E+00	0.00E+00	1.10E-05	1.55E-06
18	3.27E-05	7.49E-04	5.20E-04	1.18E-04	8.40E-06	1.14E-06
20	0.00E+00	1.09E-04	0.00E+00	0.00E+00	4.16E-06	5.30E-07
21	2.26E-05	2.08E-04	2.29E-04	8.72E-05	0.00E+00	0.00E+00
24	9.04E-05	6.48E-04	5.18E-04	1.31E-04	3.89E-05	4.43E-06
27	1.38E-05	6.42E-05	6.60E-05	1.11E-04	0.00E+00	0.00E+00
30	8.20E-05	8.44E-05	7.72E-04	4.80E-04	5.65E-05	7.53E-06
33	0.00E+00	3.98E-05	1.21E-04	5.20E-05	0.00E+00	0.00E+00
36	5.86E-05	1.40E-04	4.19E-04	8.21E-04	5.78E-05	6.89E-06
42	0.00E+00	5.10E-05	6.30E-04	5.02E-04	0.00E+00	0.00E+00
48	0.00E+00	0.00E+00	4.54E-04	7.62E-04	0.00E+00	0.00E+00
54	0.00E+00	0.00E+00	3.19E-04	6.63E-04	0.00E+00	0.00E+00
60	0.00E+00	0.00E+00	6.03E-04	2.78E-03	0.00E+00	0.00E+00
66	0.00E+00	0.00E+00	1.06E-04	1.09E-03	0.00E+00	0.00E+00
72	0.00E+00	0.00E+00	5.18E-04	1.20E-03	0.00E+00	0.00E+00
96	0.00E+00	0.00E+00	3.21E-04	2.97E-03	0.00E+00	0.00E+00
**Total Mass:**	**5.47E-04**	**5.58E-03**	**1.31E-02**	**1.24E-02**	**2.28E-04**	**2.72E-05**
**Proportion:**	**2%**	**17%**	**41%**	**39%**	**1%**	**0%**

Collection System	PVC	Vitrified Clay	Concrete	Reinforced Concrete	Cement-Lined Ductile Iron	Cement for Lining
	UEV of Material (sej/kg)					
	8.14E+13	2.82E+12	1.37E+12	1.37E+12	1.71E+13	2.75E+12
	Emergy per Volume of Water per Year ($sej/(m^3yr)$)					
**Nominal Pipe Size (in)**	**PVC**	**Vitrified Clay**	**Concrete**	**Reinforced Concrete**	**Cement-Lined Ductile Iron**	**Cement for Lining**
8	1.13E+10	2.31E+09	1.18E+09	3.14E+07	2.73E+08	4.75E+06
10	3.56E+07	3.70E+08	7.37E+07	2.99E+06	2.08E+07	3.50E+05
12	6.58E+09	5.53E+09	8.53E+09	7.16E+08	5.84E+08	9.11E+06
15	2.18E+09	1.63E+09	5.60E+08	1.09E+08	0.00E+00	0.00E+00
16	0.00E+00	0.00E+00	0.00E+00	0.00E+00	1.89E+08	4.25E+06
18	2.66E+09	2.11E+09	7.14E+08	1.62E+08	1.44E+08	3.13E+06
20	0.00E+00	3.07E+08	0.00E+00	0.00E+00	7.11E+07	1.46E+06
21	1.84E+09	5.87E+08	3.14E+08	1.20E+08	0.00E+00	0.00E+00
24	7.35E+09	1.83E+09	7.10E+08	1.80E+08	6.66E+08	1.22E+07
27	1.12E+09	1.81E+08	9.06E+07	1.52E+08	0.00E+00	0.00E+00
30	6.67E+09	2.38E+08	1.06E+09	6.58E+08	9.66E+08	2.07E+07
33	0.00E+00	1.12E+08	1.65E+08	7.13E+07	0.00E+00	0.00E+00
36	4.77E+09	3.94E+08	5.75E+08	1.13E+09	9.89E+08	1.89E+07
42	0.00E+00	1.44E+08	8.64E+08	6.89E+08	0.00E+00	0.00E+00
48	0.00E+00	0.00E+00	6.23E+08	1.05E+09	0.00E+00	0.00E+00
54	0.00E+00	0.00E+00	4.38E+08	9.09E+08	0.00E+00	0.00E+00
60	0.00E+00	0.00E+00	8.27E+08	3.81E+09	0.00E+00	0.00E+00
66	0.00E+00	0.00E+00	1.45E+08	1.50E+09	0.00E+00	0.00E+00
72	0.00E+00	0.00E+00	7.11E+08	1.64E+09	0.00E+00	0.00E+00
96	0.00E+00	0.00E+00	4.41E+08	4.08E+09	0.00E+00	0.00E+00
**Total Emergy:**	**4.45E+10**	**1.57E+10**	**1.80E+10**	**1.70E+10**	**3.90E+09**	**7.48E+07**
**Proportion:**	**45%**	**16%**	**18%**	**17%**	**4%**	**0%**

Note: Green and red shading indicate relative magnitude of emergy distributions; green shading is a comparison by size and material, red shading is a comparison of material totals.

Table S11. Basin characteristics for Lick Run UWS emergy analysis.

Parameter	Value	Source
2011 GCWW Abstraction (m^3/yr)	1.47E+08	EPA, 2014a
2011 GCWW Delivered to Consumer (m^2)	1.24E+08	EPA, 2014a
GCWW Number People Served	723,527	Communication with GCWW Staff
GCWW Per Capita Annual Use (m^3/p·yr)	171	Calculated
2011 MSDGC Treatment (m^3/yr)	1.58E+08	EPA, 2014b
2011 MSDGC Discharge (m^3/yr)	1.34E+08	EPA, 2014b
MSDGC Number of People Served	518,000	Communication with MSDGC Staff
MSDGC Per Capita Annual Production (m^3/p·yr)	304	Calculated, includes stormwater
Lick Run Population (p)	13,750	EPA, 2011, p. 3-1
Total System Area (m^2)	1.10E+07	EPA, 2011, p. 2-1
Impervious Area (m^2)	3.35E+06	EPA, 2011, p. 2-8
Vegetated Area (m^2)	7.66E+06	Vegetated area, total area minus impervious surface (EPA, 2011 p. 2-8)
Annual Precipitation (m/yr)	1.05	EPA, 2011, p. 3-15
DWTP Emergy Input, Treatment Plant (sej/m^3)	8.82E+11	This Study
DWTP Emergy Input, Distribution System (sej/m3)	8.97E+11	This Study
WWTP Emergy Input, Collection System (sej/m3)	1.79E+11	This Study
WWTP Emergy Input, Treatment Plant (sej/m3)	7.29E+11	This Study

Table S12. Lick Run urban water system emergy analysis.

Item	Description	Flow (m^3/yr)	Flow (Mm^3/yr)	Value	Unit	UEV (sej/unit)	Diagram	Emergy (sej/yr)	E+17 sej	UEV Source
Renewable Inputs										
1	Sunlight			5.50E+16	J	1		5.50E+16	0.5	Definition
2	Wind			3.11E+14	J	1.00E+03		3.11E+17	3.1	Brown and Ulgiati, 2016
3	Rain	1.16E+07	11.6	1.16E+07	m^3	3.31E+10	0.033	3.83E+17	3.8	Brown and Ulgiati, 2016
4	Ohio River	2.79E+06	2.8	2.79E+06	m^3	1.00E+11	0.10	2.79E+17	2.8	Brown and Ulgiati, 2016
Nonrenewable Inputs										
5	DW Treatment Emergy		NA	2.07E+18	sej			2.07E+18	21	This study
6	DW Distribution Emergy		NA	2.11E+18	sej			2.11E+18	21	This study
7	Food Consumption		NA	1.38E+04	cap	7.64E+15	7640	1.05E+20	1050	Johansson et al., 2000
8	WW Collection Emergy		NA	6.66E+17	sej			6.66E+17	6.7	This study
9	WW Treatment Emergy		NA	2.71E+18	sej			2.71E+18	27	This study
Internal Flows										
10	DW Distribution	2.35E+06	2.3	2.35E+06	m^3	1.90E+12	1.9	4.46E+18	45	This study
11	DW Indoor Potable	3.66E+05	0.37	3.66E+05	m^3	1.90E+12	1.9	6.94E+17	6.9	This study
12	DW Indoor Nonpotable	5.97E+05	0.60	5.97E+05	m^3	1.90E+12	1.9	1.13E+18	11	This study
13	DW Outdoor Use	1.39E+06	1.4	1.39E+06	m^3	1.90E+12	1.9	2.63E+18	26	This study
14	Indoor Effluent	9.63E+05	0.96	9.63E+05	m^3	1.11E+14	111	1.07E+20	1069	This study
15	Stormwater	1.29E+07	12.9	1.29E+07	m^3	2.33E+11	0.23	3.01E+18	30	This study
16	Evapotranspiration	4.98E+06	5.0	4.98E+06	m^3	2.33E+11	0.23	1.16E+18	12	This study
17	Groundwater Recharge	4.08E+06	4.1	4.08E+06	m^3	2.33E+11	0.23	9.49E+17	9.5	This study
18	Surface Runoff	3.88E+06	3.9	3.88E+06	m^3	2.33E+11	0.23	9.04E+17	9.0	This study
19	CSO	1.14E+06	1.1	1.14E+06	m^3	2.24E+13	22	2.54E+19	254	This study
20	WWTP Inflow	3.71E+06	3.7	3.71E+06	m^3	2.24E+13	22	8.30E+19	830	This study
21	Groundwater Discharge	4.52E+06	4.5	4.52E+06	m^3	3.97E+11	0.40	1.80E+18	18	This study
22	WWTP Discharge	3.71E+06	3.7	3.71E+06	m^3	2.31E+13	23	8.57E+19	857	This study
23	DWTP/Distribution Losses	4.46E+05	0.45	4.46E+05	m^3	1.90E+12	1.9	8.47E+17	8.5	This study
							**Total:**	**4.32E+20**		

Table S12 Notes

1 Sunlight (J/yr) = Insolation (kWh/m2/d) * Area (m2) * 3.6E+06 (J/kWh) * 365 (d/yr)

Insolation:	3.8	kWh/m^2/d	Assume average insolation for Ohio, http://solarinsolation.org/
Area:	1.10E+07	m^2	EPA, 2011, p. 2-1
Sunlight:	5.50E+16	J	
UEV:	1	sej/J	Definition

2 Wind (J/yr) = 0.5 * ρ_{air} (kg/m3) * C_D * Geostrophic Velocity^3 (m^3/s^3) * Area (m2) * 3.15E07 (s/yr)

ρ_{air}:	1.23	kg/m^3	Assuming standard temperature and pressure
C_D:	1.64E-03		Geostrophic drag coefficient over land, Garratt, 1977
Reference Velocity:	4	m/s	http://www.nrel.gov/gis/images/30m_US_Wind.jpg
Reference Elevation:	30	m	http://www.nrel.gov/gis/images/30m_US_Wind.jpg
Geostrophic Elevation:	1000	m	
Geostrophic Velocity:	9.6	m/s	Geostrophic wind over land, alpha=.25, Brown and Ulgiati, 2016
Area:	1.10E+07	m^2	EPA, 2011, p. 2-1
Wind Energy:	3.11E+14	J/yr	

Table S12 Notes

UEV:	1.00E+03	sej/J	Brown et al., 2016, 12.0E24 sej/yr baseline

3 Rain (m^3/yr) = Annual Rain (m/yr) * Area (m^2)

Annual Rain:	1.05	m/yr	EPA, 2011, p. 3-15
Area:	1.10E+07	m^2	EPA, 2011, p. 2-1
Rain:	1.16E+07	m^3/yr	
UEV:	3.31E+10	sej/m^3	Brown et al., 2016, 12.0E24 sej/yr baseline

4 Ohio River Abstraction (m^3/yr) = 1.19 * Per Capita Use (m^3/p·yr) * Capita (p)

Abstraction Factor:	1.19		1.19 m^3 abstracted for every 1 m^3 distributed
Per Capita Use:	171	m^3/p·yr	Table S13
Capita:	13750	p	EPA, 2011, p. 3-1
Ohio River Abstraction:	2.79E+06	m^3/yr	
UEV:	1.00E+11	sej/m^3	Brown and Ulgiati, 2016, 12.0E24 sej/yr baseline

5 DW Treatment Emergy (sej/yr) = Ohio River Abstraction (m^3/yr) * DWTP Unit Emergy (sej/m^3) / 1.19

Ohio River Abstraction:	2.79E+06	m^3/yr	See item 4
DWTP Unit Emergy:	8.82E+11	sej/m^3	This study
Abstraction Facter:	1.19		Normalized to cubic meter of water delivered to consumer
DW Treatment Emergy:	2.07E+18	sej/yr	

6 DW Distribution Emergy (sej/yr) = Ohio River Abstraction (m^3/yr) * Distribution Unit Emergy (sej/m^3) / 1.19

Ohio River Abstraction:	2.79E+06	m^3/yr	See item 4
Distribution Unit Emergy:	8.97E+11	sej/m^3	This study
Abstraction Facter:	1.19		Normalized to cubic meter of water delivered to consumer
DW Distribution Emergy:	2.11E+18	sej/yr	

7 Food Consumption (sej/yr) = Population (cap) * UEV (sej/cap·yr)

Lick Run Population:	13750	cap	EPA, 2011, p. 3-1
UEV:	7.64E+15	sej/cap·yr	UEV Source: Johansson et al., 2000. Adjusted from 9.44E24 sej/yr baseline
Food Consumption:	1.05E+20	sej/yr	

8 WW Collection Emergy (sej/yr) = WWTP Inflow (m^3/yr) * Collection Unit Emergy (sej/m^3)

WWTP Inflow:	3.71E+06	m^3/yr	See Item 21
Collection Unit Emergy:	1.79E+11	sej/m^3	This study
WW Collection Emergy:	6.66E+17	sej/yr	

9 WW Treatment Emergy (sej/yr) = WWTP Inflow (m^3/yr) * WWTP Unit Emergy (sej/m^3)

WWTP Inflow:	3.71E+06	m^3/yr	See Item 21
WWTP Unit Emergy:	7.29E+11	sej/m^3	This study
WW Treatment Emergy:	2.71E+18	sej/yr	

10 DW Distribution (m^3/yr) = Per Capita Annual Use (m^3/p·yr) * Lick Run Population (p)

GCWW Per Capita Annual Use:	171	(m^3/p·yr)	See Table S13
Lick Run Population:	13750	cap	EPA, 2011, p. 3-1
DW Distribution:	2.35E+06	m^3/yr	

Table S12 Notes

UEV:	1.90E+12	sej/m^3	Sum of Emergy for Items 4, 5 and 6 divided by DW Distribution volume

11 DW Indoor Potable (m^3/yr) = DW Distribution (m^3/yr) * 0.41 * 0.38

DW Distribution:	2.35E+06	m^3/yr	See Item 10.
Indoor Percentage:	0.41		41% of total DW is indoor. Mayer et al., 1999
Potable Percentage:	0.38		38% of indoor use is potable. Mayer et al., 1999
DW Indoor Potable:	3.66E+05	m^3/yr	
UEV:	1.90E+12	sej/m^3	See Item 10.

12 DW Indoor Nonpotable (m^3/yr) = DW Distribution (m^3/yr) * 0.41 * 0.62

DW Distribution:	2.35E+06	m^3/yr	See Item 10.
Indoor Percentage:	0.41		41% of total DW is indoor. Mayer et al., 1999
Nonpotable Percentage:	0.62		62% of indoor use is nonpotable. Mayer et al., 1999
DW Indoor Nonpotable:	5.97E+05	m^3/yr	
UEV:	1.90E+12	sej/m^3	See Item 10.

13 DW Outdoor Use (m^3/yr) = DW Distribution (m^3/yr) * 0.59

DW Distribution:	2.35E+06	m^3/yr	See Item 10.
Outdoor Percentage:	0.59		59% of total DW is indoor. Mayer et al., 1999
DW Outdoor Use:	1.39E+06	m^3/yr	
UEV:	1.90E+12	sej/m^3	See Item 10.

14 Indoor Effluent (m^3/yr) = DW Indoor Potable (m^3/yr) + DW Indoor Nonp Assume negligible losses in household.

DW Indoor Potable:	3.66E+05	m^3/yr	See Item 11.
DW Indoor Nonpotable:	5.97E+05	m^3/yr	See Item 12.
Indoor Effluent:	9.63E+05	m^3/yr	
UEV:	1.11E+14	sej/m^3	Sum of Emergy for Items 7, 11 and 12 divided by Indoor Effluent volume

15 Stormwater (m^3/yr) = Rain (m^3/yr) + Outdoor Use (m^3/yr)

Rain:	1.16E+07	m^3/yr	See Item 3.
DW Outdoor Use:	1.39E+06	m^3/yr	See Item 13.
Stormwater:	1.29E+07	m^3/yr	
UEV:	2.33E+11	sej/m^3	Sum of Emergy for Items 3 and 13 divided by Stormwater volume.

16 Evapotranspiration (m^3/yr) = Evapotranspiration Rate (%) * Vegetated Fraction (%) * Stormwater (m^3/yr)

Evapotranspiration Rate:	55	%	Approximately 55% of incoming water, Sanford and Selnick, 2013
Vegetated Fraction:	70	%	See Table S13
Stormwater:	1.29E+07	m^3/yr	Sum of rain and DW outdoor use
Evapotranspiration:	4.98E+06	m^3/yr	
UEV:	2.33E+11	sej/m^3	Same as Item 15.

17 Groundwater Recharge (m^3/yr) = Stormwater (m^3/yr) - Surface Runofff (m3/yr) - Evapotranspiration (m3/yr)

Stormwater:	1.29E+07	m^3/yr	See Item 15.
Surface Runoff:	3.88E+06	m^3/yr	See Item 18.
Evapotranspiration:	4.98E+06	m^3/yr	See Item 16.

Table S12 Notes

Groundwater Recharge:	4.08E+06	m^3/yr	
UEV:	2.33E+11	sej/m^3	Same as Item 15.

18 Surface Runoff (m^3/yr) = Stormwater (m^3/yr) * Impervious Fraction (%)

Stormwater:	1.29E+07	m^3/yr	See Item 15.
Impervious Fraction	30	%	See Table S13
Surface Runoff:	3.88E+06	m^3/yr	
UEV:	2.33E+11	sej/m^3	Same as Item 15.

19 CSO (m^3/yr) = Annual Overflow (gal/yr) / [7.48 (gal/m^3) * 35.3 (ft^3/m^3)]

Annual Overflow:	3.00E+08	gallon/yr	Communication with MSD
CSO:	1.13E+06	m^3/yr	
UEV:	2.24E+13	sej/m^3	Sum of Emergy for Items 8, 14 and 18, split by volume with 20, divided by CSO volume.

20 WWTP Inflow (m^3/yr) = Indoor Effluent (m^3/yr) + Surface Runoff (m^3/yr) - CSO (m^3/yr)

Indoor Effluent:	9.63E+05	m^3/yr	See Item 14.
Surface Runoff:	3.88E+06	m^3/yr	See Item 18.
CSO:	1.13E+06	m^3/yr	See Item 19.
WWTP Inflow:	3.72E+06	m^3/yr	
UEV:	2.24E+13	sej/m^3	Sum of Emergy for Items 8, 14 and 18, split by volume with 19, divided by WWTP Inflow volume.

21 Groundwater Discharge (m^3/yr) = Groundwater Recharge (m^3/yr) + DWTP/Distribution Losses (m^3/yr)

Groundwater Recharge:	4.08E+06	m^3/yr	See Item 17.
DWTP/Distribution Losses:	4.46E+05	m^3/yr	See Item 23.
Groundwater Discharge:	4.52E+06	m^3/yr	
UEV:	3.97E+11	sej/m^3	Sum of Emergy for Items 17 and 23 divided by Groundwater Discharge volume.

22 WWTP Discharge (m^3/yr) = WWTP Inflow (m^3/yr)

WWTP Inflow:	3.72E+06	m^3/yr	See Item 20.
WWTP Discharge:	3.72E+06	m^3/yr	
UEV:	2.31E+13	sej/m^3	Sum of Emergy for Items 9 and 20 divided by WWTP Discharge volume.

23 DWTP/Distribution Losses (m^3/yr) = Ohio River (m^3/yr) - DW Distribution (m^3/yr)

Ohio River:	2.79E+06	m^3/yr	See Item 4.
DW Distribution:	2.35E+06	m^3/yr	See Item 10.
DWTP/Distribution Losses:	4.46E+05		
UEV:	1.90E+12	sej/m^3	Same as Item 10.

Table S13. UEVs

UEV ID	Description	Unit	UEV (sej/unit)	Baseline (sej/yr)	Adjusted UEV (sej/unit)	Source
Environmental Flows						
106	River Water	m3	1.00E+11	1.20E+25	1.00E+11	Brown and Ulgiati, 2016.
Energy						
7	Hydroelectric Electricity	J	6.23E+04	9.44E+24	7.92E+04	Brown and Ulgiati, 2002
109	General Electricity	kWh	6.25E+11	9.44E+24	7.95E+11	Odum, 1996
119	General Electricity	J	1.74E+05	9.44E+24	2.21E+05	Odum, 1996
Fossil Fuels						
10	Natural gas	m3	6.80E+12	1.52E+25	5.37E+12	Brown et al., 2011
16	Diesel	m^3	6.48E+09	1.52E+25	5.12E+09	Brown et al., 2011
19	Gasoline	m^3	6.00E+15	1.52E+25	4.74E+15	Brown et al., 2011
Chemicals						
23	Acrylic acid	kg	3.55E+12	9.44E+24	4.51E+12	Arbault et al., 2013
25	Aluminum sulfate, powder, at plant	kg	1.99E+12	9.26E+24	2.58E+12	Rugani et al., 2011
27	Gaseous chlorine	kg	1.31E+13	9.26E+24	1.70E+13	Campbell & Ohrt, 2009
30	Hydrofluorosilicic acid, 24%	kg	5.78E+12	1.58E+25	4.38E+12	Rugani et al., 2011; Brandt-Williams, 2002
155	Quicklime, milled, packed, at plant	kg	9.85E+12	9.26E+24	1.28E+13	Rugani et al., 2011
26	Regenerated Activated Carbon	kg	8.54E+12	9.44E+24	1.09E+13	Arbault et al., 2013
28	Sodium hexametaphosphate, 30%	kg	5.67E+12	1.58E+25	4.30E+12	Brandt-Williams, 2002.
29	Sodium hydroxide, 50%	kg	5.66E+12	9.26E+24	7.33E+12	Campbell and Ohrt, 2009
31	Sodium hypochlorite, 15%	kg	2.59E+12	9.44E+24	3.29E+12	Arbault et al., 2013
154	Sodium hypochlorite, 15%	l	3.11E+12	9.44E+24	3.95E+12	Arbault et al., 2013
Building Materials - Concrete/Concrete Related						
37	Cement	kg	2.16E+12	9.44E+24	2.75E+12	Buranakarn, 1998
44	Concrete	kg	1.81E+12	1.58E+25	1.37E+12	Pulselli et al., 2008
42	Limestone, crushed, washed	kg	5.50E+12	9.26E+24	7.13E+12	Rugani et al., 2011
36	Sand	kg	1.12E+12	9.44E+24	1.42E+12	Odum, 1996, 2000
51	Transite	kg	2.07E+12	9.44E+24	2.63E+12	Brown and Buranakarn, 2003
59	Aluminum	kg	6.44E+13	9.44E+24	8.19E+13	Almeida et al., 2010
52	Cast iron, at plant	kg	1.32E+13	9.26E+24	1.71E+13	Rugani et al., 2011
58	Chromium steel 18/8, at plant	kg	1.01E+14	9.26E+24	1.31E+14	Rugani et al., 2011
50	Copper, SX-EW, at refinery	kg	1.18E+14	9.26E+24	1.53E+14	Rugani et al., 2011
56	Electrical Steel	kg	9.81E+13	9.26E+24	1.27E+14	Rugani et al., 2011
55	Reinforcing Steel	kg	1.10E+13	9.26E+24	1.43E+13	Rugani et al., 2011
57	Stainless 18/8 coil	kg	1.01E+14	9.26E+24	1.31E+14	Rugani et al., 2011
53	Steel	kg	1.48E+13	9.44E+24	1.88E+13	Buranakarn, 1998
Building Materials - Plastics/Plastics Related						
125	HDPE granulate + Extrusion	kg	4.14E+12	9.26E+24	5.37E+12	Rugani et al., 2011
66	Polyvinylchloride, at regional storage	kg	6.47E+13	9.26E+24	8.38E+13	Rugani et al., 2011
Building Materials - General						
49	Bricks	kg	2.22E+12	9.44E+24	2.82E+12	Buranakarn, 1998
39	Excavation/Earthwork	m^3	7.30E+11	9.26E+24	9.46E+11	Rugani et al., 2011
Transportation/Heavy Equipment						

Table S13. UEVs

UEV ID	Description	Unit	UEV (sej/unit)	Baseline (sej/yr)	Adjusted UEV (sej/unit)	Source
73	Barge Transport	tkm	7.27E+10	9.44E+24	9.24E+10	Buranakarn, 1998.
76	Transport, freight, rail	tkm	1.55E+11	9.26E+24	2.01E+11	Rugani et al., 2011
75	Transport, lorry 28t	tkm	6.28E+11	9.26E+24	8.14E+11	Rugani et al., 2011
Labor						
85	Labor	$	2.50E+12	1.52E+25	1.97E+12	NEAD

References

Almeida, C., Rodrigues, A.J.M., Bonilla, S.H., Giannetti, B.F., 2010. Emergy as a tool for Ecodesign: evaluating materials selection for beverage packages in Brazil. Journal of Cleaner Production 18, 32-43.

Arbault, D., Rugani, B., Tiruta-Barna, L., Benetto, E., 2013. Emergy evaluation of water treatment processes. Ecological Engineering 60, 172-182.

Brandt-Williams, S., 2002. Folio 4: emergy of Florida agriculture (2nd printing). Handbook of Emergy Evaluation. Center for Environmental Policy, Environmental Engineering Science, University of Florida, Gainesville, FL, USA

Brown, M.T., Buranakarn, V., 2003. Emergy indices and ratios for sustainable material cycles and recycle options. Resources Conservation and Recycling 38, 1-22.

Brown, M.T., Protano, G., Ulgiati, S., 2011. Assessing geobiosphere work of generating global reserves of coal, crude oil, and natural gas. Ecological Modelling 222, 879-887.

Brown, M.T., Ulgiati, S., 2002. Emergy evaluations and environmental loading of electricity production systems. Journal of Cleaner Production 10, 321-334.

Brown, M. T., Ulgiati, S., 2016. The Geobiosphere Emergy Baseline: A Synthesis. Ecological Modelling 339, 10 92-25.

Buranakarn, V., 1998. Evaluation of recycling and reuse of building materials using the emergy analysis method. University of Florida, Gainesville, FL.

Campbell, D., Ohrt, A., 2009. Environmental Accounting Using Emergy: Evaluation of Minnesota. United States Environmental Protection Agency.

Cohen, M.J., Sweeney, S., Brown, M.T., 2007. Computing the Unit Emergy Value of Crustal Elements, in: Brown, M.T. (Ed.), Emergy Synthesis 4: Theory and Applications of the Emergy Methodology. University of Florida Center for Environmental Policy, Gainesville, FL.

Garratt, J.R., 1977. DRAG COEFFICIENTS OVER OCEANS AND CONTINENTS. Monthly Weather Review 105, 915-929.

Johansson, S., Doherty, S., Rydberg, T., 2000. Sweden Food System Analysis, in: Brown, M.T. (Ed.), Emergy Synthesis: Theory and Applications of the Emergy Methodology. University of Florida Center for Environmental Policy, Gainesville, FL.

Mayer, P.W., DeOreo, W.B., Opitz, E.M., Kiefer, J.C., Davis, W.Y., Dziegielewski, B., Nelson, J.O., 1999. Residential end uses of water. AWWA Research Foundation and American Water Works Association Denver, CO.

Odum, H. T., 1996. Environmental Accounting: Emergy and Decision Making. John Wiley and Sons, New York.

Odum, H.T., 2000. Emergy of Global Processes, Folio# 2, Handbook of Emergy Evaluation. Center for Environmental Policy, Environmental Engineering Sciences, Univ. of Florida, Gainesville.

Pulselli, R.M., Sirnoncini, E., Ridolfi, R., Bastianoni, S., 2008. Specific emergy of cement and concrete: An energy-based appraisal of building materials and their transport. Ecological Indicators 8, 647-656.

Rugani, B., Huijbregts, M.A.J., Mutel, C., Bastianoni, S., Hellweg, S., 2011. Solar Energy Demand (SED) of Commodity Life Cycles. Environmental Science & Technology 45, 5426-5433.

Sanford, W.E., Selnick, D.L., 2013. Estimation of Evapotranspiration Across the Conterminous United States Using a Regression With Climate and Land-Cover Data. Journal of the American Water Resources Association 49, 217-230.

Sweeney, S., Cohen, M. J., King, D. M., Brown, M. T., 2007. Creation of a Global Emergy Database for Standardized National Emergy Synthesis. In (ed. M.T. Brown), Proceedings of the 4th Biennial Emergy Research Conference. Center for Environmental Policy, Gainesville, FL.

USEPA, 2011. Lick Run Watershed Strategic Integration Plan, Cincinnati OH. Partnership for Sustainable Communities.

USEPA, 2013. National Primary Drinking Water Regulations.

USEPA, 2014a. Environmental and Cost Life Cycle Assessment of Disinfection Options for Municipal Wastewater Treatment, in: Development, O.o.R.a. (Ed.). United States Environmental Protection Agency.

USEPA, 2014b. Environmental and Cost Life Cycle Assessment of Disinfection Options for Municipal Drinking Water Treatment in: Development, O.o.R.a. (Ed.).
